# The genome-wide binding profile of the *Sulfolobus solfataricus* transcription factor Ss-LrpB shows binding events beyond direct transcription regulation

**DOI:** 10.1186/1471-2164-14-828

**Published:** 2013-11-25

**Authors:** Trong Nguyen-Duc, Liesbeth van Oeffelen, Ningning Song, Gholamreza Hassanzadeh-Ghassabeh, Serge Muyldermans, Daniel Charlier, Eveline Peeters

**Affiliations:** Research group of Cellular and Molecular Immunology, Vrije Universiteit Brussel, Pleinlaan 2, B-1050 Brussels, Belgium; Department of Structural Biology, VIB, Pleinlaan 2, B-1050 Brussels, Belgium; Research group of Microbiology, Vrije Universiteit Brussel, Pleinlaan 2, B-1050 Brussels, Belgium; IMEC, Kapeldreef 75, B-3001 Leuven, Belgium; Department of Microbiology and Immunology, State University of New York at Buffalo, 321 Cary Hall, 3435 Main Street, 14214 Buffalo, NY United States

**Keywords:** Archaea, *Sulfolobus*, Leucine-responsive regulatory protein, CRISPR, ChIP-chip

## Abstract

**Background:**

Gene regulatory processes are largely resulting from binding of transcription factors to specific genomic targets. Leucine-responsive Regulatory Protein (Lrp) is a prevalent transcription factor family in prokaryotes, however, little information is available on biological functions of these proteins in archaea. Here, we study genome-wide binding of the Lrp-like transcription factor Ss-LrpB from *Sulfolobus solfataricus.*

**Results:**

Chromatin immunoprecipitation in combination with DNA microarray analysis (ChIP-chip) has revealed that Ss-LrpB interacts with 36 additional loci besides the four previously identified local targets. Only a subset of the newly identified binding targets, concentrated in a highly variable IS-dense genomic region, is also bound *in vitro* by pure Ss-LrpB. There is no clear relationship between the *in vitro* measured DNA-binding specificity of Ss-LrpB and the *in vivo* association suggesting a limited permissivity of the crenarchaeal chromatin for transcription factor binding. Of 37 identified binding regions, 29 are co-bound by LysM, another Lrp-like transcription factor in *S. solfataricus*. Comparative gene expression analysis in an *Ss-lrpB* mutant strain shows no significant Ss-LrpB-mediated regulation for most targeted genes, with exception of the CRISPR B cluster, which is activated by Ss-LrpB through binding to a specific motif in the leader region.

**Conclusions:**

The genome-wide binding profile presented here implies that Ss-LrpB is associated at additional genomic binding sites besides the local gene targets, but acts as a specific transcription regulator in the tested growth conditions. Moreover, we have provided evidence that two Lrp-like transcription factors in *S. solfataricus*, Ss-LrpB and LysM, interact *in vivo*.

**Electronic supplementary material:**

The online version of this article (doi:10.1186/1471-2164-14-828) contains supplementary material, which is available to authorized users.

## Background

Transcription factors (TFs) belonging to the Leucine-responsive Regulatory Protein (Lrp) family (also known as AsnC or FFRP) are abundant in both bacteria and archaea [1–4]*.* A sequence analysis of 52 archaeal genomes indicated that they are all predicted to contain at least one *lrp*-like gene, *lrp*-like genes constituting in total about 8% of all non-general TF genes in archaea [4].

Whereas bacterial Lrp-like TFs regulate amino acid biosynthesis in response to nutritional availability [5], archaeal Lrp members also regulate genes belonging to energy, central metabolism and transport pathways [6–9]. Furthermore, it has been observed and/or predicted by sequence analyses that a subset of archaeal Lrp-like TFs do not interact with amino acids, in contrast to most other archaeal Lrp-like TFs [10–16] and to bacterial Lrp-like regulators that invariably bind amino acids. Known archaeal Lrp-like TFs have regulon sizes ranging from one or a few targets to a large number of genes and operons. Examples of the former are LrpA from *Pyrococcus*[17], LrpA1 from *Halobacterium salinarum* R1 [15], Ptr2 from *Methanocaldococcus jannaschii*[6, 7] and LysM from *Sulfolobus solfataricus* that has an intermediate number of target genes [16]. Examples of the latter are FL11 from *Pyrococcus* OT3 [12], Lrp from *H. salinarum* R1 [15] and Sa-Lrp from *Sulfolobus acidocaldarius*[18].

Lrp-like proteins generally have low sequence identities, but are structurally highly conserved [19]. Typically, an Lrp monomer has a molecular mass of about 15 kDa and consists of two domains: an amino-terminal DNA-binding domain with a helix-turn-helix (HTH) motif and a carboxy-terminal domain, named Regulation of Amino acid Metabolism (RAM) [20], which is responsible for protein multimerization and cofactor binding [8]. This RAM domain forms an αβ sandwich fold having an antiparallel β sheet composed of four strands “sandwiched” between two α helices. It has been observed that, *in vitro*, archaeal Lrp-like proteins associate into several multimeric forms via β strand exchange in the RAM domain [10, 17, 21–28]. Oligomerization is a prerequisite for formation of the cofactor binding pocket. Furthermore, cofactor binding induces conformational changes that in turn could affect oligomerization [11, 13].

In *S. solfataricus*, a crenarchaeal model organism, three Lrp-like TFs have been studied experimentally: LysM [10, 16], Ss-Lrp [25] and Ss-LrpB [9, 27, 29], the latter being one of the best characterized Lrp-like regulators in archaea. Ss-LrpB performs both positive and negative autoregulation in a concentration-dependent manner [30]. Moreover, gene expression analysis in an *Ss-lrpB* deletion strain demonstrates that Ss-LrpB acts as an activator on its neighbouring target operon/genes encoding a pyruvate ferredoxin oxidoreductase (*porDAB*) and two putative permeases (*Sso2126*, *Sso2127*) [9].

At its target promoter regions, Ss-LrpB binds either a single or multiple, regularly spaced, binding sites harbouring a conserved motif [9, 29]. Each binding site is contacted by an Ss-LrpB dimer [27]. In the control region of its own gene (*Sso2131*), three Ss-LrpB dimers bind cooperatively to juxtaposed sites [31]. Occupation of all three sites results in strong DNA deformations and even DNA wrapping [27]. Based on the 15-bp palindromic consensus sequence 5'-TTGCAAAATTTGCAA-3', the sequence specificity of Ss-LrpB binding was analyzed by saturation mutagenesis [32].

Despite extensive knowledge of the *in vitro* DNA-binding properties of Ss-LrpB, nothing is known yet about its *in vivo* binding behaviour and furthermore, it is unclear whether Ss-LrpB is a local or global acting TF. In this work, we investigate Ss-LrpB binding in an *in vivo* context by performing chromatin immunoprecipitation combined with DNA microarray analysis (ChIP-chip). Besides merely identifying *in vivo* binding sites, we perform an extensive comparative analysis of *in vitro, in vivo* and *in silico* binding, exploiting the knowledge of the DNA-binding specificity model. By combining *in vivo* binding data with gene expression analysis, we provide new insights into the biological functions of Ss-LrpB, which go beyond direct transcription regulation.

## Methods

### Strains and culture conditions

*S. solfataricus* P2 (DSM1617), PBL2025 [33] and *Ss-lrpB::lacS*[9] strains were grown aerobically at 80°C in Brock basic medium supplemented with 0.1% tryptone as a carbon and energy source [34]. *Escherichia coli* strain DH5α was used for all cloning and plasmid propagation purposes. *E. coli* strain BL21(DE3) was used as a host for protein overexpression.

### Chromatin immunoprecipitation

Each chromatin immunoprecipitation (ChIP) sample was prepared from a 200 ml culture of *S. solfataricus* P2 at mid-exponential growth phase. The entire ChIP procedure, from collecting cells to obtaining amplified enriched and input DNA ready to use for microarray hybridization was performed as described [35]. In contrast to our previous work, in which a single ChIP sample was analyzed [35], we prepared and analyzed three biological replicate Ss-LrpB-specific ChIP samples. Prior to microarray hybridization analysis, samples were analyzed for enrichment relative to input DNA, which is total DNA extracted before immunoprecipitation, by quantitative PCR (qPCR) with primers specific for the *Ss-lrpB* control region (Additional file 1: Dataset S1). Furthermore, after ChIP-chip, enrichment of newly discovered binding regions was quantified similarly by qPCR. All primers are listed in Additional file 2: Table S1. qPCR was performed with a My-iQ Single Colour Real-Time PCR System (Bio-Rad) as described before [35], in triplicate and normalized to reference DNA, a non-related sequence fragment amplified from *E. coli* gDNA and spiked at 30 ng/sample before sonication.

### Microarray hybridization and data analysis

Microarray hybridizations were performed with customized 385 K high-density tiling arrays by NimbleGen (Roche) as described previously [35]. ChIP input and output samples were labelled with Cy3 and Cy5, respectively. Each probe occured twice on each array, yielding technical duplicate measurements for all samples. Microarray data analysis was performed using an extended version of the program described by Toedling and Huber [36], which uses the Ringo package of R-Bioconductor. The source code of the extended program is available via http://micr.vub.ac.be. It includes importing the data, data quality assessment, preprocessing of the data and identifying ChIP-enriched regions in a similar way as described in [16], with a threshold of 1 on the normalized log2 ratios. ChIP-enriched regions were selected as being co-associated when a LysM binding region overlapped at least partially with an extended Ss-LrpB ChIP-enriched region.

### Binding motif predictions

Using a binding energy based position weight matrix of Ss-LrpB [32], binding motifs were predicted over (i) the entire *S. solfataricus* P2 genome sequence, (ii) the genomic regions comprising 200 bp upstream of the ORF start, and (iii) the ChIP-enriched regions. The latter was also performed using the MEME suite [37]. Corresponding theoretical binding dissociation equilibrium constants (K_D_s) were calculated as well.

### DNA manipulations

Genomic DNA (gDNA) was prepared from a 10 ml *S. solfataricus* P2 culture grown until late exponential growth phase as described before [25]. Plasmid DNA was extracted from *E. coli* DH5α strains using a miniprep kit (Qiagen). For cloning of promoter regions, PCRs were performed with the FastStart High Fidelity PCR System (Roche Applied Sciences), *S. solfataricus* gDNA as a template and oligonucleotides (Sigma-Aldrich) as listed in Additional file 2: Table S1. In case of the *gpT-1/mtaP* promoter region, the oligonucleotides contained BamHI and PstI restriction sites, allowing subsequent cloning into the ampicillin resistant vector pUC18 [38]. In case of the *Sso0049* promoter region, the fragment was cloned into the pCR2.1-TOPO vector by using a TOPO TA cloning kit (Invitrogen). Individual binding sites were cloned in pBend2 by annealing two complementary oligonucleotides and ligating them into XbaI-restricted vector.

### Electrophoretic mobility shift and footprinting assays

Recombinant non-tagged Ss-LrpB and LysM were overexpressed in *E. coli* BL21(DE3) and purified as described previously [16, 27]. Electrophoretic Mobility Shift Assays (EMSAs) were performed with gel-purified 5’-end ^32^P-labelled PCR fragments generated by using ReadyMix Taq PCR Mix (Sigma-Aldrich). For validation of *in vitro* binding to *in vivo* identified binding regions, *S. solfataricus* P2 gDNA was used as a template, whereas for study of *in vitro* binding to the promoter regions and individual sites of *mtaP* and *Sso0049*, plasmid DNA was used as a template. The sequences of all oligonucleotides (Sigma-Aldrich) are provided in Table S1 (Additional file 2). The experiments were performed as described previously [22] using LrpB binding buffer [27]. The K_D_ value for binding to the CRISPR4 target was obtained using the Densitometric Image Analysis Software, available at http://micr.vub.ac.be. DNase I and ‘in gel’ copper-phenantroline (Cu-OP) footprinting assays were executed as described [22, 29]. Reference ladders were generated by chemical sequencing [39].

### Quantitative reverse transcription PCR (qRT-PCR)

For qRT-PCR analysis, 2 ml of an exponentially grown *S. solfataricus* PBL2025 or *Ss-lrpB::lacS* culture was mixed with 4 ml RNAprotect Bacteria Reagent (Qiagen) and centrifuged. Pelleted cells were subsequently lysed and RNA was extracted with the SV Total RNA Isolation System (Promega). To prevent gDNA contamination, RNA samples were treated with DNase I using the TURBO DNA-free kit (Invitrogen) according to manufacturer’s instructions. First-strand cDNA was synthesized from 1 μg RNA with SuperScript III First-Strand Synthesis SuperMix kit (Invitrogen). Primers (Additional file 2: Table S1) were designed with Primer3 software [40] and purchased at Sigma-Aldrich. qPCR was performed in a Bio-Rad iCycler with each reaction mixture containing 12.5 μl iQ SYBR Green Supermix (Bio-Rad), forward and reverse primers and 1 μl 100-fold diluted cDNA. The amplification protocol was as follows: initial denaturation at 95°C during 3 minutes, 40 cycles of 95°C during 10 seconds and 55°C during 30 seconds and one cycle of 95°C during 1 minute and 55°C during 1 minute. The melt curve analysis demonstrated absence of primer-dimer formation. All qRT-PCR assays were carried out in technical duplicate for at least four biological replicates and with a no-template and a no-RT control. Quantification cycles (C_q_s) were determined with Bio-Rad iQ5 software and mean relative gene expression ratios including standard deviations were calculated with the 2(−ΔΔC_t_) method [41]. Normalization was with respect to the expression of *tbp*, which was comparable in both strains. Data were subjected to t-test analysis using the statistical package Prism 6.0 (GraphPad).

## Results

### Genome-wide binding profile of Ss-LrpB

**Figure 1:**
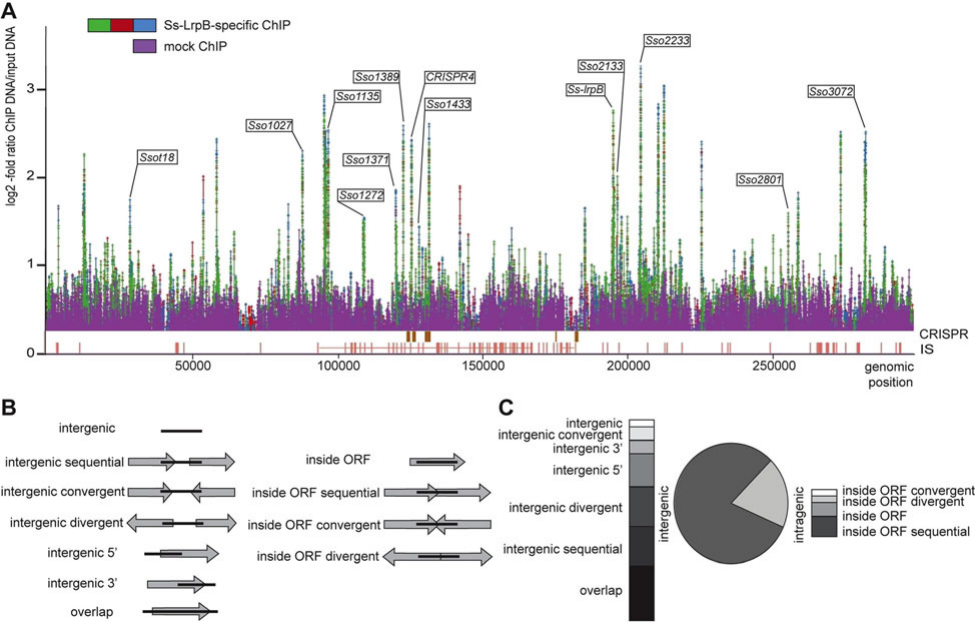
**Genome-wide distribution of Ss-LrpB binding regions. A**. Ss-LrpB binding profile generated by ChIP-chip experiments across the entire *S. solfataricus* P2 genome. These experiments were performed using an Ss-LrpB specific nanobody® (three replicate experiments), and an irrelevant nanobody® (single mock ChIP-chip experiment), according to the colour code as indicated. *S. solfataricus* P2 genomic coordinates are mentioned on the horizontal axis. Underneath plotted profiles, genomic locations of Clustered Regularly Interspaced Short Palindromic Repeats (CRISPRs) (gold bars) and IS elements (red bars) are shown, as extracted from the UCSC archaeal genome browser [56]. Targets that exhibit Ss-LrpB binding *in vitro*, are indicated. **B**. Schematic overview of location categories in which ChIP-enriched regions can be classified with respect to genomic organization. ChIP-enriched regions are indicated by black horizontal bars, whereas ORFs are depicted by horizontal arrows. **C**. Classification of Ss-LrpB binding regions in location categories as mentioned in panel B. The central pie chart shows the portions of ChIP-enriched regions overlapping at least partially with an intergenic region (dark grey) and ChIP-enriched regions that are exclusively located in intragenic regions (light grey). Stacked column charts show further division into sub-categories of peaks overlapping intergenic and coding sequences, shown on the left and right side of the pie chart, respectively.

To study genome-wide association of Ss-LrpB *in vivo*, we applied nanobody®-based ChIP-chip on exponentially growing *S. solfataricus* cells. Previously, we have used Ss-LrpB-specific nanobodies® as a proof of principle for the utilization of this class of antibodies in ChIP technology [35]. In contrast to this first study, involving a single immunoprecipitation, we now performed replicate experiments with three independent biological samples. In addition, a nanobody® recognizing a target that is absent in *S. solfataricus* cells was used in a mock ChIP experiment. Only regions exhibiting more than 2-fold enrichment in ChIP *versus* input DNA in all three Ss-LrpB-specific ChIP replicates, but not in the mock ChIP sample, were considered to be bound significantly to Ss-LrpB. In total 37 genomic regions, distributed over the entire genome, were identified (Figure 1A; Additional file 1: Dataset S1). Enrichment fold ratios ranged from 2.2 to 10.8 and were further validated by qPCR experiments, which generally yielded higher enrichment fold ratios than DNA microarray analysis, demonstrating a higher sensitivity. Nevertheless, both datasets exhibit a positive correlation (Additional file 3: Figure S1).

The genes that are overlapping or closest to the 37 ChIP peaks belong to various functional classes such as amino acid metabolism, energy metabolism, central metabolism and transport (Additional file 4: Figure S2). We also classified peaks according to their location with respect to open reading frames (ORFs) (Figure 1B and C; Additional file 1: Dataset S1). Although the majority of identified ChIP peaks are overlapping or located in an intergenic region (81%), many of these locations are unusual for a typical TF and are distant from promoters, as also demonstrated by the large distances between most peak centers and the closest experimentally determined transcription start sites (TSSs) [42] (Additional file 1: Dataset S1). Several peaks, classified as “overlap” , (partially) cover two or more ORFs (Additional file 5: Figure S3).

### *In vitro* analysis of Ss-LrpB binding to *in vivo* identified binding regions

**Figure 2:**
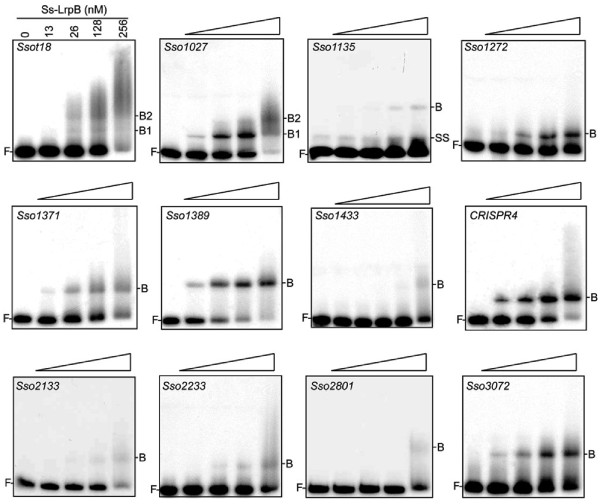
***In vitro*** **binding analysis to fragments representing ChIP-enriched genomic regions.** Overview of EMSAs that yield specific, fast migrating complexes. Targets are named according to the gene closest to or overlapping the ChIP peak (see Additional file 1: Supplementary Dataset S1). The EMSA for the *Sso2131* target is not shown as this interaction has been studied before [29]. Protein concentrations, given in the upper left EMSA, are identical for all EMSAs except for *Sso1433*, in which an additional concentration is tested (640 nM). Positions of free DNA (F), Ss-LrpB-DNA complexes (B, B1, B2) and single stranded DNA (SS) are indicated.

We performed an EMSA screen for all 36 newly identified Ss-LrpB-bound genomic regions to verify whether these target regions also interact with purified protein *in vitro* (Figure 2A; Additional file 6: Figure S4). Fragment sizes ranged from 200 to 700 bp and were designed to contain the best potential Ss-LrpB binding motif, predicted either using the binding energy weighted position matrix [32] or with the MEME suite (Additional file 1: Dataset S1). Twelve fragments displayed a concentration-dependent formation of one or two specific nucleoprotein complexes (Figure 2A) (Additional file 7: Table S2). The fast relative mobility of these complexes suggests that they contain one or maximally two Ss-LrpB dimers, rather than multiple cooperatively binding protein molecules. EMSAs performed with the other 24 fragments did not show any binding, or were indicative of unstable and nonspecific low-affinity binding resulting in smearing and/or the formation of higher-order complexes that remain in the well (Additional file 6: Figure S4).

It is notable that most of the newly identified Ss-LrpB binding sites that are directly bound, both *in vitro* and *in vivo*, are located in a highly variable region of the genome comprising multiple insertion sequence (IS) elements (Figure 1A). Furthermore, most of these Ss-LrpB binding sites are in the direct neighbourhood of an IS element. For all 12 *in vitro* bound Ss-LrpB targets, binding occurs with a lower affinity as compared to the control region of the *Ss-lrpB* gene itself [29] (Figure 2A). For example, the CRISPR4 target, one of the higher-affinity targets, is bound with an equilibrium dissociation constant (K_D_) of 63 ± 5 nM. The predictive power of the binding energy weight model appears to be limited as several predicted binding motifs have low theoretical K_D_s but nevertheless did not exhibit a specific interaction *in vitro* and *vice versa*, as several specifically bound motifs have high theoretical K_D_s (Additional file 7: Table S2).

### *In vivo* binding to *Ss-lrpB*, *porDAB*, *Sso2126* and *Sso2127* operators

**Figure 3:**
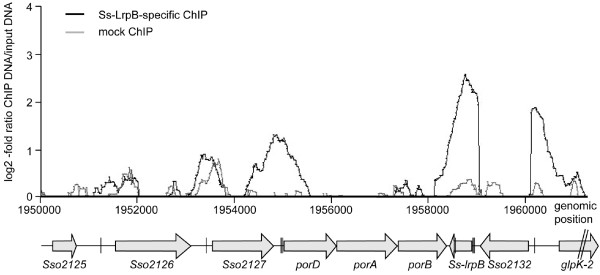
**Zoomed average ChIP-chip binding profile in the** ***Ss-lrpB*** **(** ***Sso2131*** **) genomic region.** Below the profile, relative locations of genes and characterized Ss-LrpB binding sites [9, 29] are shown in alignment with genomic positions. ORFs are indicated as horizontal arrows, whereas Ss-LrpB binding sites are depicted as vertical lines.

Upon zooming into the profile at the *Ss-lrpB* genomic region containing the regulatory target genes identified previously [9], we observed binding at all target promoters although signals in the *Sso2126*, *Sso2127* and *porDAB* promoter regions did not reach the 2-fold enrichment threshold level in all replicates (Figure 3). Averaged peak heights appear to correlate with both *in vitro* binding affinity and number of binding sites in the respective promoter/operator (p/o) regions (binding affinities/number of binding sites can be ranked as follows: p/o*Ss-lrpB* > p/o*porDAB* > p/o*Sso2127* > p/o*Sso2126*[9, 29]). Furthermore, for the targets *porDAB*, *Sso2126* and *Sso2127* this correlation can be extended to the level of activation [9].

An active copy of ISC1078 (containing a transposase encoded by *Sso2132*) is located downstream of *Ss-lrpB* with respect to genome sequence orientation [9, 43]. However, the steep decline in ChIP enrichment for the probes representing the concerned IS sequence (Figure 3) and further PCR analysis (Additional file 8: Figure S5) demonstrated the absence of this element in a large subpopulation of the cells that were subjected to ChIP.

Interestingly, the EMSA screen also resulted in a further unraveling of the *Ss-lrpB* operator for autoregulation: a fragment comprising the sequence between the insertion site of ISC1027 and the *Sso2133* (*glpK-2*) promoter results in the formation of a single specific complex (Figure 2A, *Sso2133* target). This observation implies sequence-specific recognition of another Ss-LrpB binding motif, located downstream (with respect to genome sequence orientation) of Box3 with a center-to-center distance of 174 bp (Additional file 9: Figure S6). Possibly, this site, referred to as Box6, is an auxiliary operator site that supports binding to the main high-affinity operator sites, besides the intragenic Box4 and Box5, which were identified previously as secondary operator sites (Additional file 9: Figure S6) [35].

### Ss-LrpB binds in CRISPR A and B leader regions

**Figure 4:**
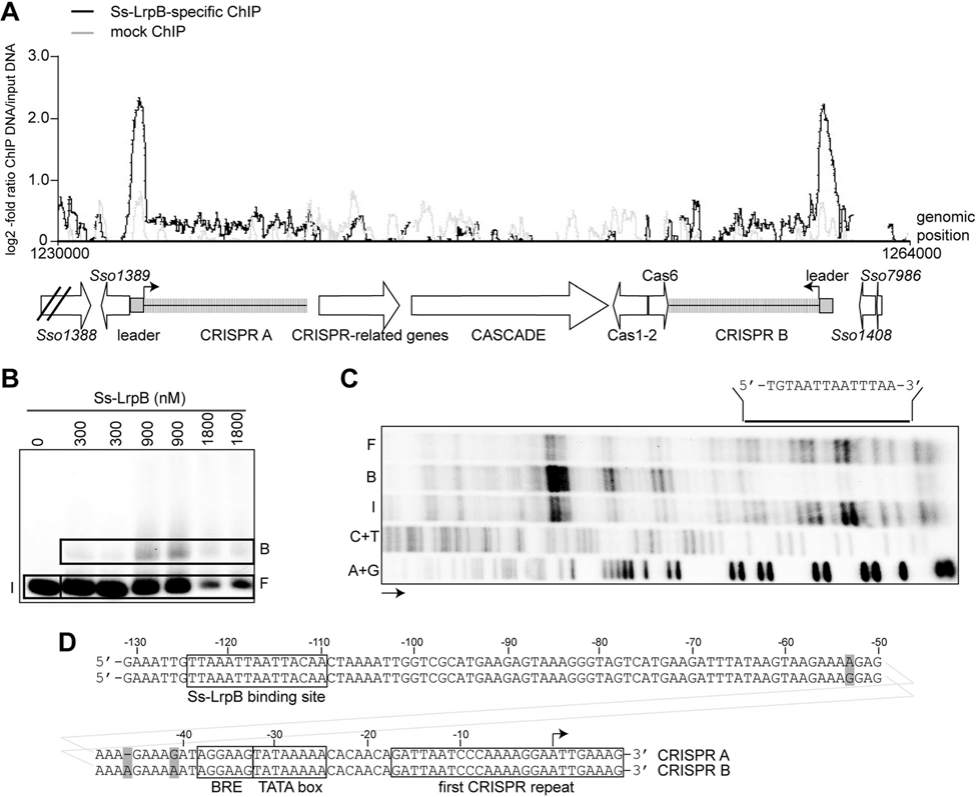
**Ss-LrpB binding to CRISPR A and CRISPR B leader regions. A**. Zoomed average ChIP-chip binding profile of the CRISPR A/B genomic region. Below the profile, the genomic organization is schematically shown aligned to genomic positions. ORFs, mainly encoding CRISPR-associated and –related genes, are indicated as horizontal arrows, whereas the leader regions are depicted as grey boxes. Primary pre-crRNA TSSs are indicated by arrows. **B**. EMSA that was used for ‘in gel’ Cu-OP footprinting, using a 270-bp fragment representing the CRISPR B leader sequence. In this experiment, the bottom strand was ^32^P-labeled. Used protein concentrations are indicated. Populations of input DNA (I), free DNA (F) and bound DNA (B) are boxed in the same way as they were excised. **C**. Autoradiograph of the denaturing gel electrophoresis of footprinting samples. Direction of electrophoresis is indicated with an arrow, lanes contain either the I, B or F populations or the A + G and C + T Maxam-Gilbert sequencing ladders, as indicated. The protected region is indicated with a horizontal line and the corresponding sequence is given (5’ - > 3’). **D**. Alignment of the promoter-proximal CRISPR A and B leader sequences, with indication of the Ss-LrpB binding site, factor B recognition element (BRE), TATA box, first CRISPR repeat and primary TSS (arrow). Non-conserved residues are highlighted in grey; position numbering is with respect to the CRISPR B leader sequence.

Ss-LrpB is associated through direct high-affinity binding with two clusters of regularly interspaced short palindromic repeats (CRISPR) loci, which are essential elements of an adaptive immunity system against viruses and other invading genetic elements. The concerned CRISPR loci, A and B, are paired family II CRISPRs sharing the same repeat sequence and are bordered by quasi-identical leader regions containing the elements to initiate and control transcription of the long pre-crRNA [44]. Ss-LrpB binding regions, previously annotated as “*Sso1389*” and “CRISPR4” , overlap the 502-bp long CRISPR A and B leader regions, respectively (Figure 4A). ‘In gel’ Cu-OP footprinting with a DNA probe representing the CRISPR B leader sequence clearly revealed Ss-LrpB-mediated protection of a stretch of about 14 nucleotides (nt) corresponding to a relatively well conserved binding motif in the promoter-proximal part of the leader (Figures 4B and C).

Given the high sequence identity between the CRISPR A and B leader regions, it is assumed that binding occurs at the corresponding site with the same sequence in the CRISPR A leader (Figure 4D). The center of the identified Ss-LrpB binding site is located 116/117 bp upstream of the main pre-crRNA TSS [42] in the first CRISPR repeat sequence, which is preceded by a strong promoter. This leads us to hypothesize that Ss-LrpB affects transcription of pre-crRNA through interaction with the basal transcription machinery.

### Permissivity of the genome for Ss-LrpB binding

Using the binding energy position weight matrix, we searched the entire *S. solfataricus* P2 genome for additional potential Ss-LrpB binding motifs. Setting the threshold for the theoretical K_D_ at 14 μM, a value still significantly lower than the average theoretical K_D_ calculated for the novel identified Ss-LrpB sites that are bound both *in vivo* and *in vitro* (290 μM), we detected 519 motifs of which 100 are located in regions 200 bp upstream of translational starts (Additional file 10: Table S3). Some of these motifs are canonical Ss-LrpB binding motifs located at appropriate distances from promoters to have the ability to exert regulation. Nevertheless, binding is absent at these locations *in vivo.*

**Figure 5:**
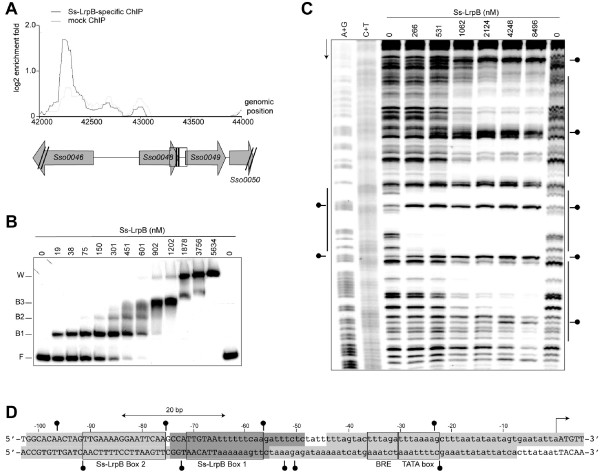
**Binding in the control region of** ***Sso0049*** **. A**. Zoomed average ChIP-chip binding profile in the genomic region of *Sso0049*. Below the profile, the genomic environment is schematically depicted by representing ORFs as grey arrows. The region corresponding to the fragment tested in *in vitro* binding analyses, is boxed, whereas the identified Ss-LrpB binding sites are represented by vertical bars. **B**. EMSA of binding to a 176 bp fragment encompassing the *Sso0049* control region. DNA populations are indicated as follows: free DNA (F), Ss-LrpB-DNA complexes (B1, B2 and B3) and complexed DNA retained in the wells of the acrylamide gel (W). Protein concentrations are given (nM). **C**. DNase I footprinting with Ss-LrpB binding to the *Sso0049* control region fragment (having the top strand ^32^P labeled). A + G and C + T represent Maxam-Gilbert sequencing reactions whereas the other lanes contain DNase I-treated samples, with indication of the applied protein concentrations. Regions that are protected against DNase I upon addition of a low Ss-LrpB concentration (< 500 nM) are indicated with a vertical line to the left-hand side of the autoradiograph, while regions that are protected at high concentration are indicated on the right-hand side. Similarly, hyperreactive sites are indicated with ball-and-stick symbols. **D**. Sequence of the *Sso0048/Sso0049* intergenic region with indication of the regions that are protected against DNase I in the experiment shown in subpanel C (dark grey = protected at all Ss-LrpB concentrations; light grey = only protected at high Ss-LrpB concentrations) and in an experiment using DNA with the bottom strand labeled. Coding sequences are shown in uppercase (left side = *Sso0048*, right side = *Sso0049*). The TSS of *Sso0049*, indicated with an arrow, was experimentally determined with primer extension analysis (Additional file 13: Figure S9).

We selected two examples to illustrate this disagreement between binding *in vitro* and *in vivo* (Figure 5; Additional file 11: Figure S7)*.* The promoter region of *Sso0049*, encoding an unknown protein, contains a canonical binding motif (5’-TTGTAATTTTTTCAA-3’) that is identical to the high-affinity Box 1 of the *Ss-lrpB* operator 5’-TTGTAATTTTTACAA-3’ with the exception of one A → T substitution at a less critical position. An EMSA probing binding of Ss-LrpB to a p/o*Sso0049* fragment revealed the formation of three distinct protein-DNA complexes (Figure 5B). DNase I footprinting showed that it is indeed the predicted binding motif referred to as Box 1 that is bound at low protein concentration and is most likely protected in complex B1 in the EMSA (Figures 5B and C). Furthermore, an EMSA using a fragment containing solely the *Sso0049* Box 1 confirmed a high-affinity interaction (K_D_ ≈ 150 nM; Additional file 12: Figure S8). At higher Ss-LrpB concentrations, DNase I protection in the p/o*Sso0049* fragment was extended both downstream and upstream of Box 1. Upon manual inspection of the sequence, we identified an additional binding motif (Box 2) with a center-to-center-distance of 20 bp upstream of Box 1 (Figure 5C). Despite the high-affinity, cooperative binding to multiple binding sites *in vitro*, no enrichment of this genomic region has been detected in the ChIP-chip assay (Figure 5A). Notably, Ss-LrpB is associated with the genome about 1 kb upstream of the *Sso0049* control region in the ORF of *Sso0046*. This observation suggests that absence of Ss-LrpB at p/o*Sso0049* is not caused by limited diffusion of the TF throughout the cell but rather to an inaccessibility of chromatin or the DNA sequence itself at this locus.

A similar discrepancy between *in vitro* and *in vivo* binding was shown to exist for the intergenic promoter region shared between the divergently transcribed *Sso2342*, encoding a purine phosphoribosyltransferase (*gpT-1*), and *Sso2343*, encoding a 5’-methylthioadenosine phosphorylase (*mtaP*) (Additional file 11: Figure S7; Additional file 12: Figure S8; Additional file 13: Figure S9). Altogether, these observations demonstrate that the target DNA sites within the *S. solfataricus* genome are not entirely permissive for Ss-LrpB binding.

### Overlap between Ss-LrpB and LysM binding profiles

**Figure 6:**
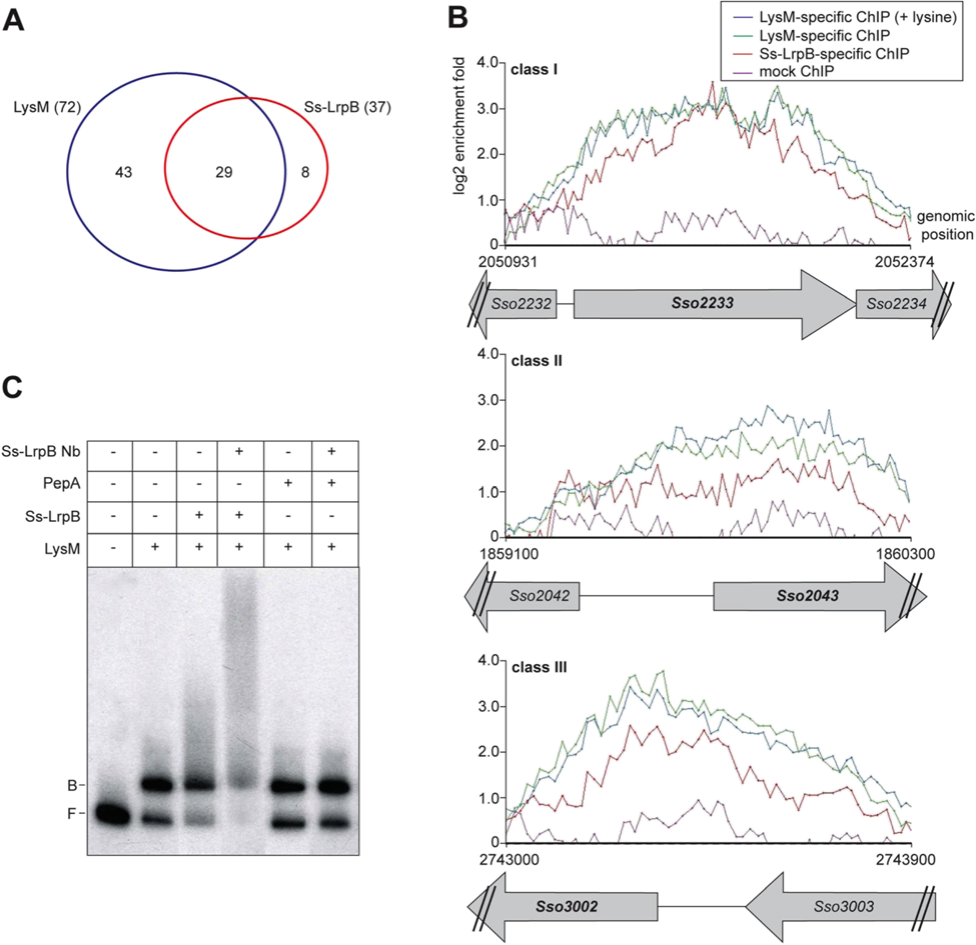
**Overlap between Ss-LrpB and LysM binding profiles. A**. Venn diagram illustrating shared binding regions between Ss-LrpB and LysM. **B**. Zoomed binding profiles of Ss-LrpB- and LysM-specific ChIP assays for three selected targeted regions, belonging to one of three classes: class I: binding region that contains an Ss-LrpB binding motif and exhibits binding to Ss-LrpB but not LysM *in vitro*; class II: binding region that contains a LysM binding motif and exhibits binding to LysM but not Ss-LrpB *in vitro*; class III: binding region that does not contain an Ss-LrpB or LysM binding motif and does not interact with any of the two proteins *in vitro*. Aligned with the binding profiles, the genomic organization is depicted by representing ORFs as arrows. **C**. EMSA demonstrating simultaneous binding of Ss-LrpB and LysM to a 348-bp probe encompassing the *Ssot28* target. The positions of free DNA (F) and bound DNA (B) are indicated. The following protein concentrations have been used: 40 nM LysM, 320 nM Ss-LrpB, 500 nM Ss-LrpB-specific Nb and 500 nM *E. coli* PepA, which was used as a negative control.

*S. solfataricus* possesses additional Lrp-like TFs, such as the lysine-responsive LysM. We compared the Ss-LrpB binding profile to the locations of the LysM binding regions mapped previously [16] and observed a significant overlap: 29 of the 37 Ss-LrpB binding regions were also associated with LysM (Figure 6A; Table 1). Of note, no cross-reactivity of Ss-LrpB- and LysM-specific nanobodies with other Lrp-type proteins has ever been observed [16, 35]. Zoomed binding profiles for both TFs are highly similar, which is a striking observation given that both profiles were recorded in different growth conditions (sucrose-supplemented for LysM-specific ChIP *versus* tryptone-supplemented medium for Ss-LrpB-specific ChIP) (Figure 6B).

**Table 1:** **Overview of the Ss-LrpB-specific ChIP-enriched regions that are also enriched in LysM-specific ChIP-chip analysis**

Target name	Genomic coordinate peak start	Genomic coordinate peak stop	Relative position to LysM-specific peak	Presence of Ss-LrpB binding motif	Presence of LysM binding motif	Class
*Sso0154*	129616	130620	Inside	-	-	III
*Sso5317*	131930	133008	Including	-	+	II
*Ssot18*	290428	291131	Inside	+	-	I
*Ssot28*	589572	590362	overlap (89%)	-	+	II
*Sso6904*	835210	835839	Inside	-	+	II
*Sso1027*	885479	886771	Inside	+	-	I
*Sso1114*	960343	961574	Including	-	-*	III
*Sso1118*	964702	966378	Including	-	-*	III
*Sso1135*	974250	975551	overlap (99%)	+	-*	I
*Sso1272*	1095398	1097098	Inside	+	-*	I
*Sso1371*	1206614	1207226	overlap (97%)	+	-*	I
*Sso1389*	1232759	1233642	overlap (99%)	+	-*	I
CRISPR4	1260282	1261337	Including	+	-*	I
*Sso1433*	1286420	1286928	Inside	+	-*	I
*Sso1463*	1321798	1323084	overlap (98%)	-	-*	III
*Sso1890*	1705187	1705830	Inside	-	-*	III
*Sso2043*	1859092	1860291	overlap (75%)	-	+	II
*Sso2159*	1985046	1985784	Inside	-	-*	III
*Sso2233*	2050931	2052374	Including	+	-*	I
*Sso2289*	2099312	2099902	Inside	-	-*	III
*Sso2309*	2111358	2112389	Inside	-	-*	III
*Sso2310*	2113136	2114378	overlap (93% )	-	-*	III
*Sso2334*	2132622	2134455	Including	-	+	II
*Sso2404*	2187776	2188318	Inside	-	-*	III
*Sso2678*	2437317	2437768	overlap (96%)	-	-*	III
*Sso2801*	2561656	2562152	overlap (57%)	-	-*	III
*Sso2833*	2594520	2595831	overlap (96%)	-	-*	III
*Sso3002*	2742947	2743947	Including	-	-*	III
*Sso3072*	2827204	2828448	overlap (94%)	+	-*	I

The presence of an Ss-LrpB binding motif (measured by EMSA) or a LysM binding motif (either measured by EMSA or predicted *in silico* (indicated by an asterisk) [16]) is indicated. Classes are defined as follows: class I: binding region that contains an Ss-LrpB binding motif and exhibits binding to Ss-LrpB but not LysM *in vitro*; class II: binding region that contains a LysM binding motif and exhibits binding to LysM but not Ss-LrpB *in vitro*; class III: binding region that does not contain an Ss-LrpB or LysM binding motif and does not interact with any of the two proteins *in vitro*. Ranking is according to genomic location.

The known regulatory targets of Ss-LrpB, *Sso2126*, *Sso2127* and *porDAB*, are not co-bound by LysM, in contrast to most of the newly discovered low-affinity Ss-LrpB binding targets. Conversely, the main local regulatory target of LysM, the *lysWXJK* operon for lysine biosynthesis, is also bound by Ss-LrpB. To distinguish between (i) a mutually exclusive binding of either Ss-LrpB or LysM at a particular target in a subpopulation of cells, or (ii) a situation where the proteins bind as a co-complex to this target, we compared *in vitro* and *in silico* binding characteristics for these targets (Table 1). Shared binding regions could be categorized in three classes: (i) binding regions that contain an Ss-LrpB binding motif and exhibit binding to Ss-LrpB but not LysM *in vitro* (class I); (ii) binding regions that contain a LysM binding motif and exhibit (predicted) binding to LysM but not Ss-LrpB *in vitro* (class II) and (iii) binding regions that do not contain an Ss-LrpB or LysM binding motif and do not interact with any of the two proteins *in vitro* (class III). There is a perfect inverse correlation pattern between the presence of a *bona fide* Ss-LrpB or LysM binding motif suggesting that the TFs are co-localized through protein-protein interaction and that only one of the protein partners directly interacts with the DNA.

We further investigated the simultaneous interaction of LysM and Ss-LrpB to one of the co-bound targets, *Ssot28*, in an *in vitro* assay (Figure 6C). Whereas Ss-LrpB does not form specific complexes with this target (Additional file 6: Figure S4), LysM forms a single complex by binding to a site located close to the promoter of the glutamate synthase (*gltB*) gene [16]. EMSA analysis demonstrated that the addition of Ss-LrpB to reaction mixtures containing LysM and the DNA stimulated slightly the complex formation (Figure 6C). Furthermore, the addition of Ss-LrpB-specific antibodies resulted in a clear supershift of the complex. These observations suggest that Ss-LrpB is present in the nucleoprotein complex, despite that it is unable to interact with the DNA fragment by itself.

### Gene expression analysis of transcripts associated with Ss-LrpB binding regions

**Figure 7:**
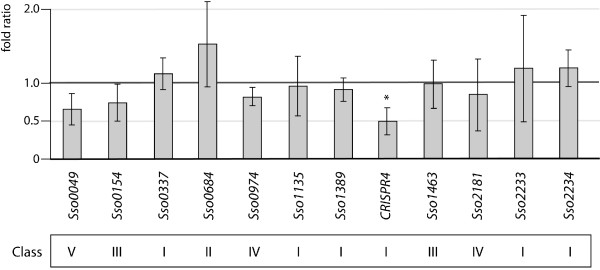
**Expression analysis of genes of which the coding regions are located close to a selection of Ss-LrpB binding regions.** Tested genes are subdivided into the following classes: class I: gene associated to binding region bound by LysM and Ss-LrpB that contains an Ss-LrpB binding motif and exhibits binding to Ss-LrpB but not LysM *in vitro*; class II: gene associated to binding region bound by LysM and Ss-LrpB that contains a LysM binding motif and exhibits binding to LysM but not Ss-LrpB *in vitro*; class III: gene associated to binding region bound by LysM and Ss-LrpB that does not contain an Ss-LrpB or LysM binding motif and does not interact with any of the two proteins *in vitro*; class IV: gene associated to binding region solely bound by Ss-LrpB *in vivo*; class V: gene associated to region that is bound by Ss-LrpB *in vitro* but not *in vivo*. Relative gene expression ratios in an *Ss-lrpB::lacS versus* PBL2025 strain are normalized against *tbp* expression. Values are the average of biological quadruplicates and standard deviations represent the biological variation. An asterisk indicates a P-value of 0.085. All other tested genes render P-values > 0.1, indicating no significant differences in expression. Absence of *Ss-lrpB* expression in the *Ss-lrpB::lacS* strain has been confirmed (fold ratio = 0.0031 ± 0.0020; P-value = 0.0001).

To determine whether the identified binding events cause transcription regulation of neighbouring genes, we investigated the effect of deleting *Ss-lrpB* on the expression of a wide selection of potential target genes by quantitative reverse transcriptase PCR (qRT-PCR) (Figure 7). Some of these genes were associated with direct Ss-LrpB binding (class I), others with indirect binding (class II, III and IV), yet others with co-binding of Ss-LrpB and LysM (class I, II and III) (for a definition of the classes, see legend of Figure 7). For some of them, binding occurs relatively close to the promoter whereas for other genes the binding is intragenic and far away from the closest promoter region (e.g. *Sso2233*). With the exception of the CRISPR B pre-crRNA, deletion of *Ss-lrpB* did not significantly affect the expression of any of these tested target genes. We have also confirmed that the TF does not significantly affect the expression of *Sso0049*, which contains high-affinity Ss-LrpB binding sites in its promoter region that are however not associated with Ss-LrpB *in vivo*.

The expression of CRISPR B pre-crRNA is moderately downregulated (2-fold) in the *Ss-lrpB::lacS* strain in comparison to the isogenic WT, indicating an Ss-LrpB-mediated activation. It is assumed that a similar regulation will be exerted at the CRISPR A locus. In conclusion, Ss-LrpB is involved in CRISPR regulation whereas the other identified binding events appear to occur without apparent regulation under our conditions of growth.

## Discussion

Our genome-wide localization study has revealed the association of Ss-LrpB with at least 37 loci in the *S. solfataricus* genome. A subset of these *in vivo* Ss-LrpB-targeted sites is clearly validated to be bound with this pure TF *in vitro* and to contain a *bona fide* sequence motif. Obviously, the well-established, high sequence specificity of Ss-LrpB [32] is responsible for the complex formation at these sites. However, computational analysis with a binding energy based position weight matrix of the *S. solfataricus* genome demonstrated a vast overrepresentation of appropriate sequence motifs of which only a very small subset seems to be actually bound *in vivo*. A high number of false negative signals in the ChIP-chip analysis, e.g. due to a too stringent threshold, is a possible explanation for the observed lack of *in vivo* binding at predicted motifs. However, a closer investigation of the binding profiles at loci containing multiple high-affinity binding motifs (promoter regions of *Sso0049* and *mtaP*) confirmed complete absence of Ss-LrpB-specific enrichment in these regions. Therefore, we conclude that the intrinsic DNA-binding sequence specificity is a poor predictor of binding *in vivo* under our culture conditions of *S. solfataricus* and that additional factors are involved in determining site selectivity and occupancy *in vivo*.

*In vivo* binding site selectivity could be influenced by the structural landscape of the chromatin that imposes differential genome accessibility on a higher organizational level. Similarly to bacteria, Crenarchaeota have their nucleoid structurally organized by small chromatin proteins [45], however it is unknown how this genome packaging affects TF binding. Possibly, Ss-LrpB binding is restricted by the action of nucleoid associated proteins resulting in differential accessible genomic regions. Apparently, accessibility is facilitated in a highly variable region of the *S. solfataricus* genome with multiple IS elements and low abundance of essential genes. However, there are alternative explanations available for the lack of association at high-affinity binding motifs. For instance, it might be caused by the presence of a co-repressor, ligand or post-translational modification that inhibits Ss-LrpB binding under the used culture conditions or, on the contrary, by the absence of a critical ligand *in vivo* that activates binding and was present in the *in vitro* binding reaction mixtures, possibly through co-purification after heterologous expression of Ss-LrpB in *E. coli*. Technical details, such as unstable behavior of the TF-DNA complexes during formaldehyde fixation or sonication, could also lead to certain binding events not being detected.

There is a very low correlation between Ss-LrpB binding and transcription regulation, which appears to be limited to the *Sso2126*, *Sso2127* and *porDAB* gene targets that were identified previously and to the CRISPR A and B clusters. Furthermore, a significant fraction of binding regions is located at a significant distance from the nearest TSS and associated promoter and/or is even intragenic, an observation made for bacterial [46–49] and for archaeal TFs as well [16, 50]. The newly discovered genomic targets that contain a binding motif are generally contacted by Ss-LrpB through non-cooperative low-affinity binding, in contrast to the local gene targets. Low-affinity binding without apparent regulation appears to be universal as it has been observed repeatedly for TFs of *M. tuberculosis*[51], yeast [52] and *Drosophila*[53]. However, the biological function of these weak binding sites is still unclear. It has been hypothesized that they could display a subtle regulatory activity, which goes undetected and causes a fine-tuning of gene regulation. In this way, these binding sites alleviate selective pressure on specific loci, namely classical regulatory binding sites, and increase the ability to evolve [53]. An alternative explanation for the biological role of these low-affinity sites is that they might create a biological buffering system that serves as a reservoir to sequester TF molecules, thereby thermodynamically regulating the concentration of freely available protein [53, 54]. This could be a critical factor for correct functioning of Ss-LrpB, given the limited number of target genes and the cooperative nature of the interaction at these targets.

The observed occupancy levels and regulatory effects of the major regulatory targets (*Sso2126*, *Sso2127*, *porDAB*[9] and CRISPR B) are weak and were most probably recorded in a growth condition yielding a non-regulated “ground state” . Possibly, a different, as yet unknown, culturing condition leads to higher occupation and corresponding activation levels of the regulatory target genes. Similarly, some of the newly identified binding sites might mediate regulation of nearby genes under different growth conditions than those in which the binding profile was monitored. The exact functions and substrate specificities of the pyruvate ferredoxine oxidoreductase and the two permeases are unclear although it is tentative to speculate that these proteins function during lactate oxidation or a related metabolic pathway [9]. Indeed, *Sso2126* encodes a permease that exhibits homology with bacterial L-lactate permeases and *Sso2127* codes for a homolog of halophilic oxalate/formate antiporters. Protein sequence analysis suggests that if Ss-LrpB is bound by an effector molecule, it is a small molecule other than an amino acid [9].

The growth condition that is more relevant for Ss-LrpB regulation might be a more stressful condition for the cells, given the Ss-LrpB-mediated CRISPR activation. It has been demonstrated that expression of CRISPR and CRISPR-associated (CAS) genes is inducible by abiotic and/or biotic stress [55]. The high energetic cost of expressing the long pre-crRNA leads to hypothesize that it is under a complex transcriptional regulation involving multiple regulators, of which we demonstrate that Ss-LrpB is the first promoter-interacting TF to be characterized in *S. solfataricus*. Co-regulation of *porDAB*, *Sso2126* and *Sso2127* on one hand and the CRISPR clusters on the other hand indicates that there is a possible link between metabolic regulation and immunity defense in *S. solfataricus*.

Remarkably, the Ss-LrpB and LysM binding profiles display a significant overlap. The detection of the two proteins at the same genomic location in different ChIP-chip profiles could be explained by (i) binding of a hetero-protein complex of LysM and Ss-LrpB to the DNA target or (ii) a heterogeneous occupancy where Ss-LrpB is present on the DNA site in some cells, and LysM on the corresponding DNA site in other cells or (iii) a combination of both possibilities. In the case of these two Lrp-like TFs, the inverse correlation pattern between the presence of a LysM or Ss-LrpB binding motif suggests that they are simultaneously associated as a complex with the target DNA sites in the *S. solfataricus* genome. Furthermore, this co-association occurs most likely via protein-protein interactions in which only one of the protein partners interacts with DNA, rather than by cooperative interactions between distinct DNA-bound TF molecules. This proposal is supported by our *in vitro* experiments where Ss-LrpB was shown to be present in the protein-DNA complex although it does not interact with the DNA by itself. For a distinct class (“class III”) of binding targets, direct DNA recognition by Ss-LrpB or LysM was clearly absent, suggesting the involvement of additional TFs. Remarkably, we did not observe significant Ss-LrpB-mediated regulation of the major LysM targets to which co-association was observed. Possibly, the presence of Ss-LrpB results only in subtle regulatory effects, fine-tuning the regulatory action of LysM, and the involvement of different partners is partially interchangeable. Our data demonstrate that archaeal Lrp-like TFs interact *in vivo*, supporting previous data that *Pyrococcus* Lrp-like TFs tend to form hetero-oligomeric structures *in vitro*[11, 17].

## Conclusions

In conclusion, our genome-wide association study of Ss-LrpB yields novel insights into its *in vivo* interactions despite providing only limited additional information on its physiological role. Ss-LrpB interacts with multiple low-affinity sites throughout the genome without an apparent regulatory purpose and these sites are often associated with IS elements. Furthermore, the TF binds in the CRISPR A and B leader regions and activates expression of pre-crRNA. Ss-LrpB also co-associates with another Lrp-like TF, LysM. Hetero-oligomerization of archaeal Lrp proteins was previously observed *in vitro* and thus, we provide the first indications of an interplay of two of these factors *in vivo*. Finally, the absence of Ss-LrpB *in vivo* on sites carrying a well-predicted binding motif suggests a limited permissivity of the *S. solfataricus* genome for association with its cognate TF.

## Availability of supporting data

All the supporting data are included as additional files.

## Electronic supplementary material

Additional file 1: Dataset S1: Overview of all ChIP-enriched regions identified in the study, with indication of details of qPCR and EMSA experiments for validation. (XLSX 20 KB)

Additional file 2: Table S1: List of oligonucleotide sequences used in this work. (PDF 126 KB)

Additional file 3: Figure S1: Statistical analysis of Ss-LrpB ChIP enrichment obtained by DNA microarray hybridization and qPCR analysis. (PDF 194 KB)

Additional file 4: Figure S2: Pie chart displaying the fractions of functional classes to which the genes, closest to or overlapping the identified Ss-LrpB binding regions, belong. (PDF 1 MB)

Additional file 5: Figure S3: Zoomed profiles of ChIP-enriched regions (partially) covering two open reading frames or more. (PDF 273 KB)

Additional file 6: Figure S4: *In vitro* binding analysis to fragments representing ChIP-enriched genomic regions. (PDF 793 KB)

Additional file 7: Table S2: (Putative) binding motifs in the sequences bound by Ss-LrpB *in vivo* and *in vitro*. (PDF 73 KB)

Additional file 8: Figure S5: PCR analysis of genomic DNA targeting the genomic region that flanks *Ss-lrpB* and contains an ISC1078 element between positions 1959047 and 1960125 in the published *S. solfataricus* P2 genome sequence. (PDF 177 KB)

Additional file 9: Figure S6: Schematic representation of the *Ss-lrpB* operator structure in the case of absence of ISC1078. (PDF 174 KB)

Additional file 10: Table S3: *In silico* predicted Ss-LrpB binding sites (using the binding energy weight matrix) in regions upstream of *S. solfataricus* P2 ORFs (200 bp upstream of ORF start) with a theoretical K_D_ lower than 5 μM. (PDF 91 KB)

Additional file 11: Figure S7: *In vitro* binding in the control region of *gpT-1* (*Sso2342*)/ *mtaP* (*Sso2343*). (PDF 1 MB)

Additional file 12: Figure S8: *In vitro* binding to the separate Ss-LrpB binding sites identified in the *Sso0049* and *gpT-1*/*mtaP* control regions. (PDF 300 KB)

Additional file 13: Figure S9: Primer extension analysis for TSS determination. (PDF 274 KB)
