# Supplementary material for: The genome-wide binding profile of the Sulfolobus solfataricus transcription factor Ss-LrpB shows binding events beyond direct transcription regulation
Source: BMC Genomics. 2013 Nov 25;14(1):828. doi: 10.1186/1471-2164-14-828 (PMC4046817; doi:10.1186/1471-2164-14-828)
Supplement: Supplementary file 2 — Additional file 2: Table S1: List of oligonucleotide sequences used in this work. (PDF 126 KB) [file 12864_2013_5555_MOESM2_ESM.pdf]

Table S1. List of oligonucleotide sequences used in this work.

A. Oligonucleotides used for qPCR validation of ChIP enrichment. “-L” indicates left primer; “-R” right primer. Target regions are named according to Table 1. Ranking is according to genomic location.

Name	Sequence	Target
Sso0046-qPCR-L	5'-AATCTTTCATTCCTCCTCTTATATGG-3'	*Sso0046*
Sso0046-qPCR-R	5'-CATCCAGAATTTGTAAAGGATAAGG-3'	*Sso0046*
Sso0154-qPCR-L	5'-CCGTAGTTGGAGGTTCAGGA-3'	*Sso0154*
Sso0154-qPCR-R	5'-TGGTTTTCCCGCATATTCTC-3'	*Sso0154*
Sso5317-qPCR-L	5'-AGACGATGCGTTACCAGGAG-3'	*Sso5317*
Sso5317-qPCR-R	5'-TCACTCTCCCCAATCCTCAC-3'	*Sso5317*
Ssot18-qPCR-L	5'-AGTATGGTGGTTTTCCTTAGTGG-3'	*Ssot18*
Ssot18-qPCR-R	5'-GCCGGCCTCCTAACTTTC-3'	*Ssot18*
Sso6264-qPCR-L	5'-GGGCGATAAACTAGTAATTGGAG-3'	*Sso6264*
Sso6264-qPCR-R	5'-TTCTCCTTCTGGATCTCTAACTGAG-3'	*Sso6264*
Ssot28-qPCR-L	5'-GAGCAATCTAACCTCATGATAACTG-3'	*Ssot28*
Ssot28-qPCR-R	5'-GGTTTGATTTCCGAACTTAAGAG-3'	*Ssot28*
Ssot36-qPCR-L	5'-AGTATCTCGTTAATAACATCGTCTTTG-3'	*Ssot36*
Ssot36-qPCR-R	5'-TCAGGTCGCACTTTATTATTTAGC-3'	*Ssot36*
Sso6904-qPCR-L	5'-TGTCGAACCTTTCTTTACCG-3'	*Sso6904*
Sso6904-qPCR-R	5'-TCTCCTTCTCTTAACGTCGAATC-3'	*Sso6904*
Sso1027-qPCR-L	5'-TCCGCATGTTAATATCGAAGC-3'	*Sso1027*
Sso1027-qPCR-R	5'-TTCGATTCTATTTCCAGCCTAAG-3'	*Sso1027*
Sso1114-qPCR-L	5'-TGATTGACGGTTCTACTCAAGG-3'	*Sso1114*
Sso1114-qPCR-R	5'-ATATCGGAACATCTCCTCTCG-3'	*Sso1114*
Sso1118-qPCR-L	5'-TGAGAGTAAGAAGTCCACTTGACC-3'	*Sso1118*
Sso1118-qPCR-R	5'-AGTAGGTTATCCAAGATAGTTGACG-3'	*Sso1118*
Sso1135-qPCR-L	5'-TATCCCGTTGGAGAAAATCG-3'	*Sso1135*
Sso1135-qPCR-R	5'-GCTAATGCCAAGTTCCTTGC-3'	*Sso1135*
Sso1272-qPCR-L	5'-AAGGCTTGCCACAACTATTACC-3'	*Sso1272*
Sso1272-qPCR-R	5'-AAACGCACCGATCTCTATACC-3'	*Sso1272*
Sso1371-qPCR-L	5'-AAAGTTTTTGTCCCAGAGTCG-3'	*Sso1371*
Sso1371-qPCR-R	5'-AAAAGTTCGAGCCTCAATGC-3'	*Sso1371*
Sso1389-qPCR-L	5'-AGTCTCAATGCGACCGAAAC-3'	*Sso1389*
Sso1389-qPCR-R	5'-CCTTTACTCTTCATGCGACCA-3'	*Sso1389*
CRISPR4-qPCR-L	5'-CCTTTACTCTTCATGCGACCA-3'	*CRISPR4*
CRISPR4-qPCR-R	5'-GATGAAAGGAGAGCGATAAGAGA-3'	*CRISPR4*
Sso1433-qPCR-L	5'-TGGAGAAGATGACGAAATGG-3'	*Sso1433*
Sso1433-qPCR-R	5'-TCTAGACATTGTGGCTAAAGATAAGG-3'	*Sso1433*

Sso1463-qPCR-L	5'-ATCGACTTCAGCAACTGTCC-3'	*Sso1463*
Sso1463-qPCR-R	5'-GGCTTTTCGGGCTATCTCC-3'	*Sso1463*
Sso1890-qPCR-L	5’-TTCAGCTATCTAACTCTATGGTAGGC-3’	*Sso1890*
Sso1890-qPCR-R	5’-CGAACGCCCTTTTTCTTAGC-3’	*Sso1890*
Sso2043-qPCR-L	5'-TCAGTAACTCCGATAAGTTGTTAAGG-3'	*Sso2043*
Sso2043-qPCR-R	5'-GCCGAGAGAAGATATAGAAATGC-3'	*Sso2043*
Sso2131-qPCR-L	5'-TTGCGCATCTTCCAATCTC-3'	*Sso2131*
Sso2131-qPCR-R	5'-TTTGCGCTATAATGCTAAAAAGA-3'	*Sso2131*
Sso2159-qPCR-L	5'-TCACACGAAGCAACTATACCG-3'	*Sso2159*
Sso2159-qPCR-R	5'-CAAGAATATGGAGGTCTTTCAGC-3'	*Sso2159*
Sso2181-qPCR-L	5'-ACTCGGTGGAAAAACTATAGCC-3'	*Sso2181*
Sso2181-qPCR-R	5'-GCATTGGAAGCTCAGATATTGG-3'	*Sso2181*
Sso2233-qPCR-L	5'-TTGCTACCGGTTACCTAACTCC-3'	*Sso2233*
Sso2233-qPCR-R	5'-AAGTTCTTGCCCTCAACTGC-3'	*Sso2233*
Sso2289-qPCR-L	5'-AAAACTACTGGAAAGCCAGAGG-3'	*Sso2289*
Sso2289-qPCR-R	5'-ATTCTACCACCATCCGCAAC-3'	*Sso2289*
Sso2309-qPCR-L	5'-TGGTTTTATTATCGCGCAAG-3'	*Sso2309*
Sso2309-qPCR-R	5'-TGGATGGAAACAATGCACTAAC-3'	*Sso2309*
Sso2310-qPCR-L	5'-TGATAAAACTACCTGGTCATACGC-3'	*Sso2310*
Sso2310-qPCR-R	5'-TGTAAAAGGGAACTGAGAAACC-3'	*Sso2310*
Sso2334-qPCR-L	5'-GGCGACATCCCAACTCATAC-3'	*Sso2334*
Sso2334-qPCR-R	5'-GTAGAAACGGCGGGATAATG-3'	*Sso2334*
Sso2801-qPCR-L	5'-TTCCGTTCTTATCGAATGTGAG-3'	*Sso2801*
Sso2801-qPCR-R	5'-AAAATGAAGCAAGTAGAAGAACACC-3'	*Sso2801*
Sso2833-qPCR-L	5'-GGAAATTCGTATTTTCTTCCA-3'	*Sso2833*
Sso2833-qPCR-R	5'-TTCACCGTCTTGAGACCTAACA-3'	*Sso2833*
Sso3002-qPCR-L	5'-CCATGTTCCTTTTCCCTAAGC-3'	*Sso3002*
Sso3002-qPCR-R	5'-CTTCCAGCAAGCCGTAGTTC-3'	*Sso3002*
Sso3072-qPCR-L	5'-AGGCATATTCAATACCTTAAGCAG-3'	*Sso3072*
Sso3072-qPCR-R	5'-AAAATGGCAATCTTTCTGACG-3'	*Sso3072*
DC821	5’-CGGGATCCAGTCATTCATCGACTCATGCC-3’	*E. coli* reference DNA
DC822	5’-CGGGATCCATGCATAGCTATTCTCTTTTGTTAATTTGC-3’	*E. coli* reference DNA

B. Oligonucleotides used for EMSA fragment preparation. "-L" indicates left primer; "-R" right primer. Target regions are named according to Table 1. Ranking is according to genomic location.

Name	Sequence	Target
Sso0046-L	5'-AATCTTTCATTCCTCCTCTTATATGG-3'	*Sso0046*
Sso0046-R	5'-CATCCAGAATTTGTAAAGGATAAGG-3'	*Sso0046*
Sso0154-L	5'-AAGGCTGAAAAATATGACTTGC-3'	*Sso0154*
Sso0154-R	5'-TGGTTTTCCCGCATATTCTC-3'	*Sso0154*
Sso5317-L	5'-TACGCCACAAATACCGACAC-3'	*Sso5317*
Sso5317-R	5'-CCAATCCTCACCTATTTGCTC-3'	*Sso5317*
Ssot18-L	5'-AGTATGGTGGTTTTCCTTAGTGG-3'	*Ssot18*
Ssot18-R	5'-GCCGGCCTCCTAACTTTC-3'	*Ssot18*
Sso6264-L	5'-GGGCGATAAACTAGTAATTGGAG-3'	*Sso6264*
Sso6264-R	5'-TCCCTGGGAACTTAATTATTGC-3'	*Sso6264*
Ssot28-L	5'-AGTATGGTGGTTTTCCTTAGTGG-3'	*Ssot28*
Ssot28-R	5'-GTATAGCAGAATTCTTTTTATATATTG-3'	*Ssot28*
Ssot36-L	5'-AGTATCTCGTTAATAACATCGTCTTTG-3'	*Ssot36*
Ssot36-R	5'-TCAGGTCGCACTTTATTATTTAGC-3'	*Ssot36*
Sso0974-L	5'-TTTTTCATCTCATTTTTATGTTACGC-3'	*Sso0974*
Sso0974-R	5'-AAGCTGGTTAAATTCCTCTAATTTC-3'	*Sso0974*
Sso6904-L	5'-ATATGTATATTCAAGCCATTTAGA-3'	*Sso6904*
Sso6904-R	5'-GGATATATAATGAATTCGGTAAAGAAA-3'	*Sso6904*
Sso1027-L	5'-TCCGCATGTTAATATCGAAGC-3'	*Sso1027*
Sso1027-R	5'-TGAGCTCGTACAATAGTGATAA-3'	*Sso1027*
Sso1114-L	5'-CAGTGTGAAGGCAATTGGAA-3'	*Sso1114*
Sso1114-R	5'-CGGTAAATGGTTGCCCTAAA-3'	*Sso1114*
Sso1118-L	5'-GCGTTGAACATTGAGTCCAG-3'	*Sso1118*
Sso1118-R	5'-AAGGGCTTTGCAATTACTATCG-3'	*Sso1118*
Sso1135-L	5'-CGCATTAGAAGTAAAGAACGTAAA-3'	*Sso1135*
Sso1135-R	5'-TATTGTAAATATATTCAACAGCGTGA-3'	*Sso1135*
Sso1272-L	5'-GACTTTATTGCCAGAAATGCAA-3'	*Sso1272*
Sso1272-R	5'-TTTTCCACTGCAAACTTTTT-3'	*Sso1272*
Sso1371-L	5'-AAAGTTTTTGTCCCAGAGTCG-3'	*Sso1371*
Sso1371-R	5'-AAAAGTTCGAGCCTCAATGC-3'	*Sso1371*
Sso1389-L	5'-AACTTATTCTTAATTCTCAGATAAACG-3'	*Sso1389*
Sso1389-R	5'-TCTTCATGACTACCCTTTACTCTTCA-3'	*Sso1389*
CRISPR4-L	5'-CCTTTACTCTTCATGCGACCA-3'	*CRISPR4*
CRISPR4-R	5'-GATGAAAGGAGAGCGATAAGAGA-3'	*CRISPR4*

Sso1433-L	5'-TGGAGAAGATGACGAAATGG-3'	*Sso1433*
Sso1433-R	5'-TCTAGACATTGTGGCTAAAGATAAGG-3'	*Sso1433*
Sso1463-L	5'-TTGATGGCGTTAGTGTGACAA-3'	*Sso1463*
Sso1463-R	5'-ACTGCAATTTTCGCAATGTC-3'	*Sso1463*
Sso1890-L	5’-CAGCCATAATCCTTGTACTGATG-3’	*Sso1890*
Sso1890-R	5’-TCTGGGAATCTAATTCTTGAAGC-3’	*Sso1890*
Sso2043-L	5’-TCAGTAACTCCGATAAGTTGTTAAGG-3’	*Sso2043*
Sso2043-R	5’-GAAAACAGTAATCCGGGAAGC-3’	*Sso2043*
Sso2133-L	5’-TTGCTCTTTAAAGTCTGACGAAG-3’	*Sso2133*
Sso2133-R	5’-TGTGAATGTTTTCCTTCCCTAAG-3’	*Sso2133*
Sso2142-L	5’-TGTGAACTTGTTGAATTTTCCAG-3’	*Sso2142*
Sso2142-R	5’-TGAAGACTTCTTAAGGGCAAAAAC-3’	*Sso2142*
Sso2159-L	5’-TCACACGAAGCAACTATACCG-3’	*Sso2159*
Sso2159-R	5’-TCACACGAAGCAACTATACCG-3’	*Sso2159*
Sso2181-L	5’-TTCCCATTTAATGCTTTCTCTAGC-3’	*Sso2181*
Sso2181-R	5’-GCATTGGAAGCTCAGATATTGG-3’	*Sso2181*
Sso2233-L	5'-GGTGGTAAGATAATAGACAGAAAGGA-3'	*Sso2233*
Sso2233-R	5'-CATTGGCGATTTCATTAATAGTCTT-3'	*Sso2233*
Sso2289-L	5'-GAAAGCCAGAGGATATAGCAAA-3'	*Sso2289*
Sso2289-R	5'-ATTCTACCACCATCCGCAAC-3'	*Sso2289*
Sso2309-L	5’-ATGAACAACGACGGATTACC-3’	*Sso2309*
Sso2309-R	5’-GAGTATGAGGAGAGATTTGGATGG-3’	*Sso2309*
Sso2310-L	5’-GGACATTTTTACACCAAACTGC-3’	*Sso2310*
Sso2310-R	5’-AAGGGAACTGAGAAACCTTC-3’	*Sso2310*
Sso2334-L	5’-ACCGCCTCTACACCTTCATC-3’	*Sso2334*
Sso2334-R	5’-TTTGATGGTACTTGTGATTTATGC-3’	*Sso2334*
Sso2404-L	5’-TTGCGAATGGAAAGCCTAAG-3’	*Sso2404*
Sso2404-R	5’-ATTGCTGGCGTTTATATTGAAG-3’	*Sso2404*
Sso2678-L	5’-TCCACAATTGACTGTAGCTTCC-3’	*Sso2678*
Sso2678-R	5’-AGCCCTAGCAAAGTCCAAGC-3’	*Sso2678*
Sso2801-L	5’-TTCCGTTCTTATCGAATGTGAG-3’	*Sso2801*
Sso2801-R	5’-CAAATCCGCATTACCAACAAC-3’	*Sso2801*
Sso2833-L	5’-CAATTCGGAGTACTGAATGATGG-3’	*Sso2833*
Sso2833-R	5’-TGAGAAAGCTATGATACAACAGAGG-3’	*Sso2833*
Sso3002-L	5’-GGTTCATCACACCCATCTCC-3’	*Sso3002*
Sso3002-R	5’-CTTCCAGCAAGCCGTAGTTC-3’	*Sso3002*
Sso3072-L	5'-TGCTATTTGTGAGAAAAGATGAAA-3'	*Sso3072*
Sso3072-R	5'-GAAAGTTGGAAAAATGGCAATC-3'	*Sso3072*

C. Oligonucleotides used for qRT-PCR analysis of gene expression. “-L” indicates left primer; “-R” right primer. Ranking is according to genomic location.

Name	Sequence	Gene
ep275-L	5’-AGACGCTCTGGAATCTGAGG-3’	*Sso0049*
ep276-R	5’-TTTTCACTTTTCGCTTATCTTCG-3’	*Sso0049*
ep285-L	5’-AAATCTCGGCTAGAATTTCACC-3’	*Sso0154*
ep286-R	5’-TTACAAGCTTAAGAAAGGAATTGG-3’	*Sso0154*
DC1134-L	5’-GGAACAGACACCCTTGCACT-3’	*Sso0337*
DC1135-R	5’-ATCCATCCTAATGCCCATCC-3’	*Sso0337*
DC1223-L	5′-GAAGCGTTCAGTTGGAGTG-3′	*Sso0684*
DC1224-R	5′-GCGATACCTCTTCCATCAC-3'	*Sso0684*
ep277-L	5’-ATGTTGGCTGATGACTACGC-3’	*Sso0974*
ep278-R	5’-GGAGCATCAAACATTGTACCG-3’	*Sso0974*
DC1140-L	5’-ACCCAGAGAGGCCATTCTTT-3’	*Sso1135*
DC1141-R	5’-ACATGAGGACCACCACAACA-3’	*Sso1135*
DC1144-L	5’-GGTCGAGAGCTAAGGCAAGA-3’	*Sso1371*
DC1145-R	5’-AGCTTCTCCCCTCAAAGTCC-3’	*Sso1371*
DC1146-L	5’-ACTCCCTCCGAAAACGAAAT-3’	*Sso1389*
DC1147-R	5’-GTCTCGGCGTAGTGCCTTAG-3’	*Sso1389*
ep287-L	5’-GCTGCTGCTAATTGGTCTCC-3’	*CRISPR4*
ep288-R	5’-TCTGTGCGAGCTTGTTTACG-3’	*CRISPR4*
ep279-L	5’-GCCGGAATAGGAGAAAAAGG-3’	*Sso1463*
ep280-R	5’-AGGGACAGTTGCTGAAGTCG-3’	*Sso1463*
ep283-L	5’-ATAGACCCCGGTGAGGTAGC-3’	*Sso2181*
ep284-R	5’-TTCATTTTTCCGCCTATTGC-3’	*Sso2181*
DC1150-L	5’-TGTAAGTAAAGATGGGGAACCA-3’	*Sso2233*
DC1151-R	5’-CAGCAAGAATTGGAGCTAACG-3’	*Sso2233*
DC1152-L	5’-CCGGAAATCCATCTGAAGAA-3’	*Sso2234*
DC1153-R	5’-GGCCATGATGAGTAGCTTCC-3’	*Sso2234*

D. Oligonucleotides used for other purposes.

Name	Sequence	Purpose
IS-left	5’-GCATAAAATGAAGTTTTATATCGC-3’	Analysis genomic structure
IS-right	5’-ACGTTGTAAAATGGGCGAAC-3’	Analysis genomic structure
DC100	5’-AACTGCAGCTTAAAGGTAAGATGCTTAGC-3’	Cloning p/o *Sso0049*
DC101	5’-CGGGATCCTCTTCTCCTATGCCAAAC-3’	Cloning p/o *Sso0049*

DC102	5'-AACTGCAGCATTGACGTTATACTCATCAGC-3'	Cloning p/o *gpT-1*/*mtaP*
DC103	5'-CGGGATCCAATCATAAGATGAAATACAC-3'	Cloning p/o *gpT-1*/*mtaP*
DC179	5'-GATACCCGGATCGTATAGTCCAGATCC-3'	Primer extension *mtaP*
DC180	5'-GTCATTGGACCCTTAGATAAGACG-3'	Primer extension *Sso0049*
DC189	5'-CTAGATTGGCAAAATGTGCAAGT-3'	Cloning Box 2 p/o *Sso0049*
DC190	5'-CTAGACTTGAAAAAATTACAATT-3'	Cloning Box 2 p/o *Sso0049*
DC193	5'-CTAGAGTTAAAACTTTATCAATT-3'	Cloning Box 1 p/o *gpT-1*/*mtaP*
DC194	5'-CTAGAATTGATAAAGTTTTAACT-3'	Cloning Box 1 p/o *gpT-1*/*mtaP*
DC195	5'-CTAGACTGGTATTTTTTGCAAAT-3'	Cloning Box 2 p/o *gpT-1*/*mtaP*
DC196	5'-CTAGATTTGCAAAAAATACCAGT-3'	Cloning Box 2 p/o *gpT-1*/*mtaP*
