# Supplementary material for: The genome-wide binding profile of the Sulfolobus solfataricus transcription factor Ss-LrpB shows binding events beyond direct transcription regulation
Source: BMC Genomics. 2013 Nov 25;14(1):828. doi: 10.1186/1471-2164-14-828 (PMC4046817; doi:10.1186/1471-2164-14-828)

Figure S1. Statistical analysis of Ss-LrpB ChIP enrichment data obtained by DNA microarray hybridization and qPCR analysis. A. Boxplot representations of the distributions of log_2 fold-ratio enrichments as assessed by microarray hybridization and qPCR. The microarray-generated data (maximum log_2 values) consist of 37 data points, whereas the qPCR data (mean log_2 values) consist of 33 data points. **B.** Correlation of qPCR and microarray enrichment data. Data for 33 out of 37 ChIP-enriched regions (those validated by qPCR; Additional file 1: Supplementary Dataset S1) were correlated by linear regression analysis. The Pearson's correlation coefficient r is 0.7788.

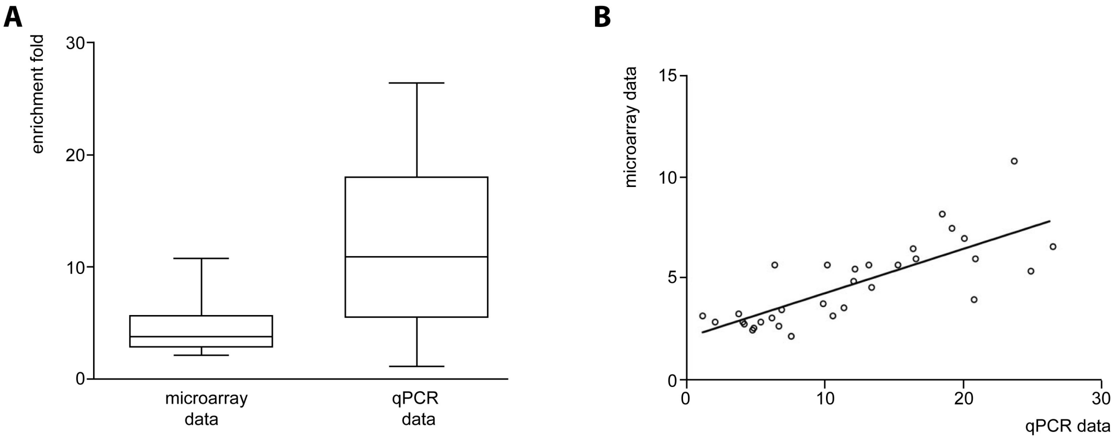

Supplement: Supplementary file 3 — Additional file 3: Figure S1: Statistical analysis of Ss-LrpB ChIP enrichment obtained by DNA microarray hybridization and qPCR analysis. (PDF 194 KB) [file 12864_2013_5555_MOESM3_ESM.pdf]
