# Supplementary material for: The genome-wide binding profile of the Sulfolobus solfataricus transcription factor Ss-LrpB shows binding events beyond direct transcription regulation
Source: BMC Genomics. 2013 Nov 25;14(1):828. doi: 10.1186/1471-2164-14-828 (PMC4046817; doi:10.1186/1471-2164-14-828)

Figure S2. Pie chart displaying the fractions of functional classes to which the genes, closest to or overlapping the identified Ss-LrpB binding regions, belong.

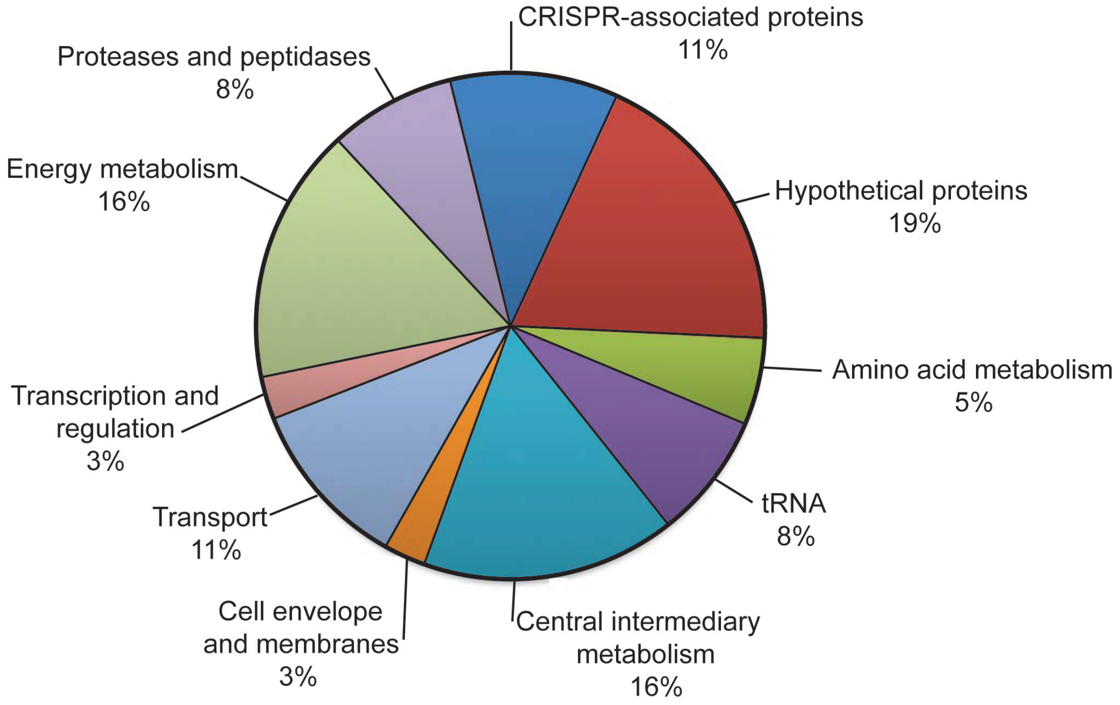

Supplement: Supplementary file 4 — Additional file 4: Figure S2: Pie chart displaying the fractions of functional classes to which the genes, closest to or overlapping the identified Ss-LrpB binding regions, belong. (PDF 1 MB) [file 12864_2013_5555_MOESM4_ESM.pdf]
