# Supplementary material for: The genome-wide binding profile of the Sulfolobus solfataricus transcription factor Ss-LrpB shows binding events beyond direct transcription regulation
Source: BMC Genomics. 2013 Nov 25;14(1):828. doi: 10.1186/1471-2164-14-828 (PMC4046817; doi:10.1186/1471-2164-14-828)

Figure S3. Zoomed profiles of ChIP-enriched regions (partially) covering two open reading frames or more. Log_2 values of fold enrichment are plotted (y-axis) *versus* genomic position (x-axis). The averaged Ss-LrpB-specific ChIP profile is plotted in black and the mock ChIP profile in grey. Sso gene numbers used for annotation of the shown ChIP-enriched region (Additional file 1: Supplementary Dataset S1) are in bold italics.

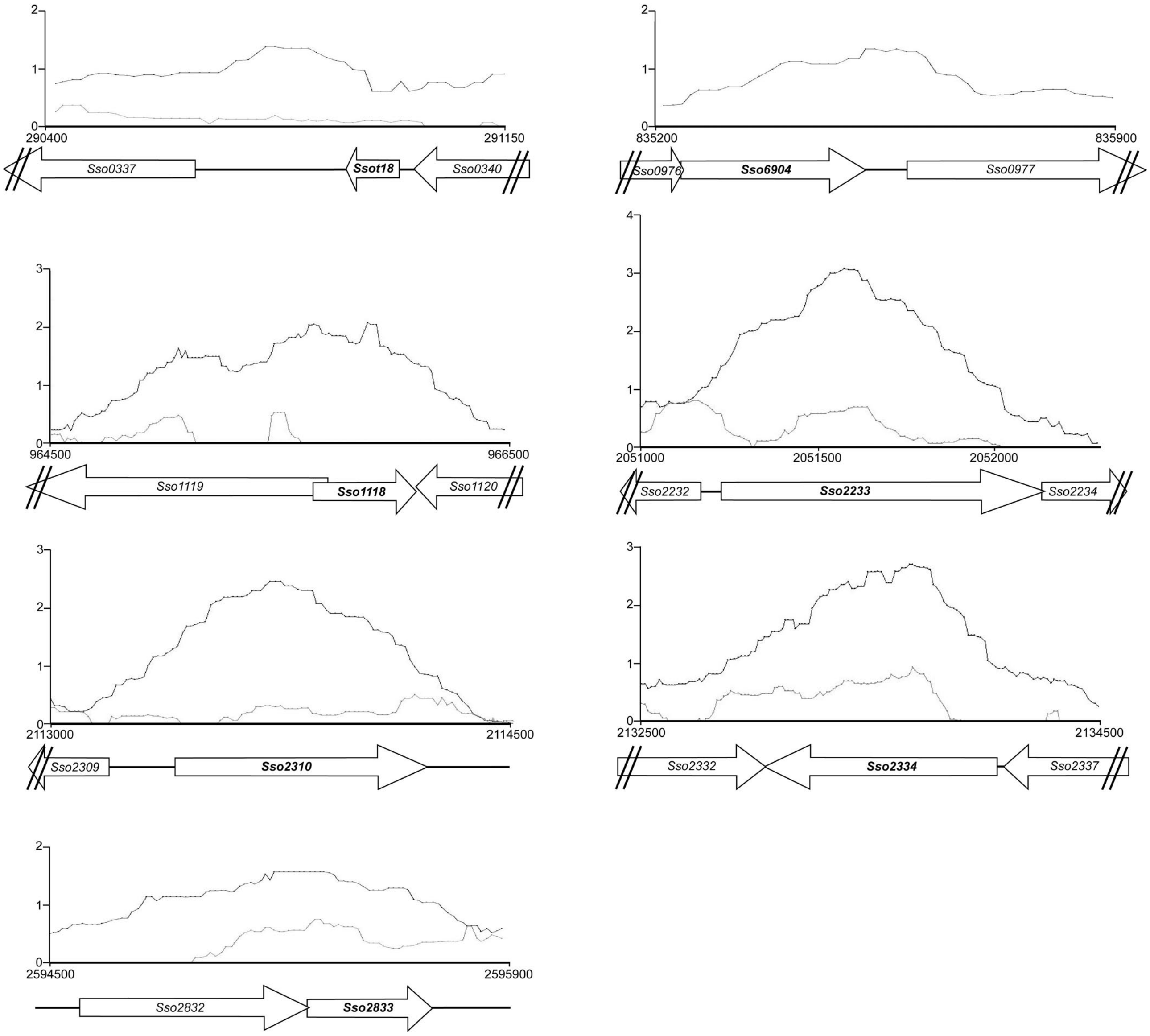

Supplement: Supplementary file 5 — Additional file 5: Figure S3: Zoomed profiles of ChIP-enriched regions (partially) covering two open reading frames or more. (PDF 273 KB) [file 12864_2013_5555_MOESM5_ESM.pdf]
