# Supplementary material for: The genome-wide binding profile of the Sulfolobus solfataricus transcription factor Ss-LrpB shows binding events beyond direct transcription regulation
Source: BMC Genomics. 2013 Nov 25;14(1):828. doi: 10.1186/1471-2164-14-828 (PMC4046817; doi:10.1186/1471-2164-14-828)

Figure S4. *In vitro* binding analysis to fragments representing ChIP-enriched genomic regions. In this figure, all EMSAs are shown that yield nonspecific, low-affinity or no binding at all for the fragments containing the best potential motif predicted with the energy based position weight matrix. Protein concentrations are identical for all EMSAs and are given in the upper left EMSA. Targets are named according to the gene closest to or overlapping the ChIP peak. Disappearance of DNA at the highest protein concentrations indicates that non-specific binding causes the material to remain in the wells.

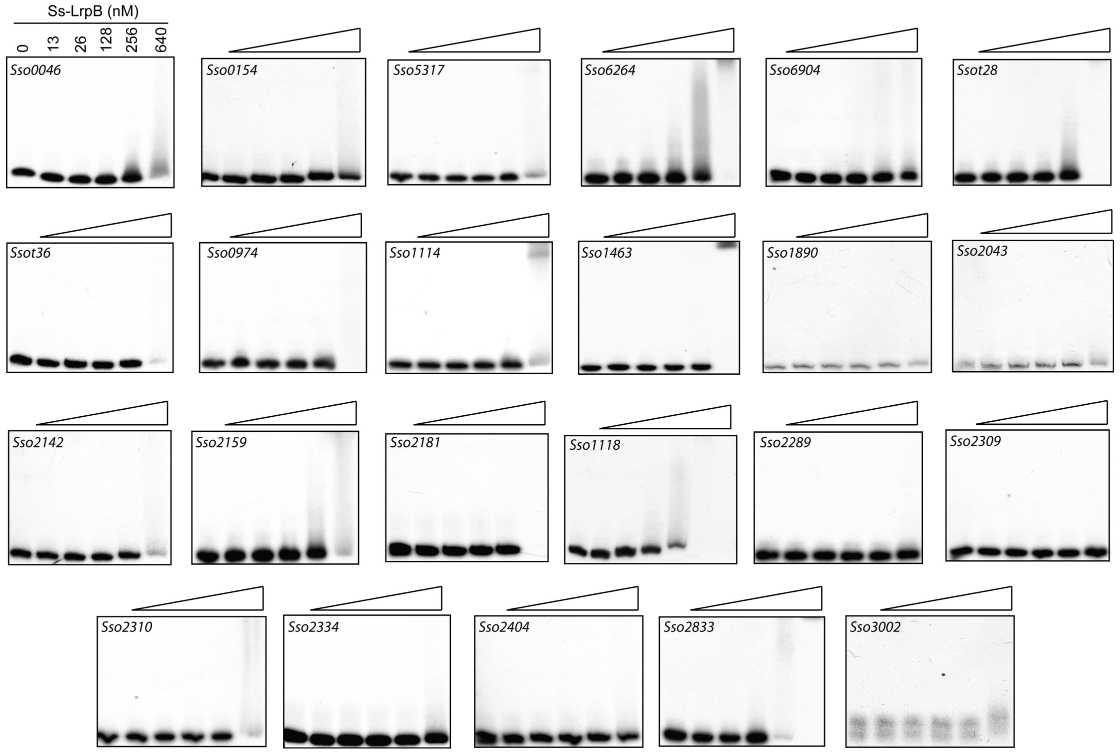

Supplement: Supplementary file 6 — Additional file 6: Figure S4: In vitro binding analysis to fragments representing ChIP-enriched genomic regions. (PDF 793 KB) [file 12864_2013_5555_MOESM6_ESM.pdf]
