# Supplementary material for: The genome-wide binding profile of the Sulfolobus solfataricus transcription factor Ss-LrpB shows binding events beyond direct transcription regulation
Source: BMC Genomics. 2013 Nov 25;14(1):828. doi: 10.1186/1471-2164-14-828 (PMC4046817; doi:10.1186/1471-2164-14-828)
Supplement: Supplementary file 7 — Additional file 7: Table S2: (Putative) binding motifs in the sequences bound by Ss-LrpB in vivo and in vitro. (PDF 73 KB) [file 12864_2013_5555_MOESM7_ESM.pdf]

Table S2. (Putative) binding motifs in the sequences bound by Ss-LrpB *in vivo* and *in vitro*. Predictions of recognized binding motifs were performed using the binding energy based position weight matrix (Peeters *et al.* 2007) (indicated by "binding energy"), MEME (indicated by "MEME") or were confirmed by experimental observations (indicated by "footprinting") (Fig. 4). In case of different predictions by the binding energy based position weight matrix and MEME, multiple fragments were tested for binding to avoid missing a binding motif. Binding affinities (expressed as K_D) are theoretical and predicted using the binding energy based position weight matrix.

**Target name**	**Binding motif sequence**	**Prediction/identification of binding motif**	**K_D (μM)**
Ssot18	5'-CAGTAATTATTATAA-3'	binding energy	290
Sso1027	5'-TTTCAAAATGTTAAA-3'	MEME	43538
Sso1135	5'-TTGTCACTATTCCTT-3'	MEME	585
Sso1272	5'-TTGCCAGAAATGCAA-3'	binding energy	6
Sso1371	5'-ATGGAAAGCTTACAT-3'	binding energy	237
Sso1389	5'-TTGTAATTAATTTAA-3'	footprinting	137
Sso1433	5'-TTGCATATAATTCCG-3'	binding energy	56
CRISPR4	5'-TTGTAATTAATTTAA-3'	footprinting	137
Sso2133	5'-TCGCCCATTTTACAA-3'	binding energy	40
Sso2233	5'-ATGATGAAATTTCTA-3'	MEME	884
Sso2801	5'-TAGCAAATGTTCCAA-3'	binding energy	3
Sso3072	5'-TTGCCATTTTTCCAA-3'	binding energy	5

Reference

Peeters E, Wartel C, Maes D, Charlier D: **Analysis of the DNA-binding sequence specificity of the archaeal transcriptional regulator Ss-LrpB from *Sulfolobus solfataricus* by systematic mutagenesis and high resolution contact probing.** *Nucleic Acids Res* 2007, **35**:623–633.
