# Supplementary material for: The genome-wide binding profile of the Sulfolobus solfataricus transcription factor Ss-LrpB shows binding events beyond direct transcription regulation
Source: BMC Genomics. 2013 Nov 25;14(1):828. doi: 10.1186/1471-2164-14-828 (PMC4046817; doi:10.1186/1471-2164-14-828)
Supplement: Supplementary file 8 — Additional file 8: Figure S5: PCR analysis of genomic DNA targeting the genomic region that flanks Ss-lrpB and contains an ISC1078 element between positions 1959047 and 1960125 in the published S. solfataricus P2 genome sequence. (PDF 177 KB) [file 12864_2013_5555_MOESM8_ESM.pdf]

Figure S5. PCR analysis of genomic DNA targeting the genomic region that flanks *Ss-lrpB* and contains an ISC1078 element between positions 1959047 and 1960125 in the published *S. solfataricus* genome P2 sequence (She *et al.*, 2001). PCRs were performed with primers IS-left and IS-right, generating a 1297 bp-fragment (B) upon presence and a 218 bp-fragment (A) upon absence of the insertion sequence (IS) element. Complete absence of the IS element is confirmed in the PBL2025 and *Ss-lrpB::lacS* strains (Peeters *et al.*, 2009), whereas the P2 strain that was used for ChIP-chip analysis is a mixed population with only a (minor) subpopulation harbouring ISC1078. This observation is in line with transposable elements, and ISC1078 in particular, being very active in *S. solfataricus* P2 (Redder & Garrett, 2006). All template PCRs were performed in duplicate. NTC = non template control; MWL = molecular weight ladder (indicated in bp).

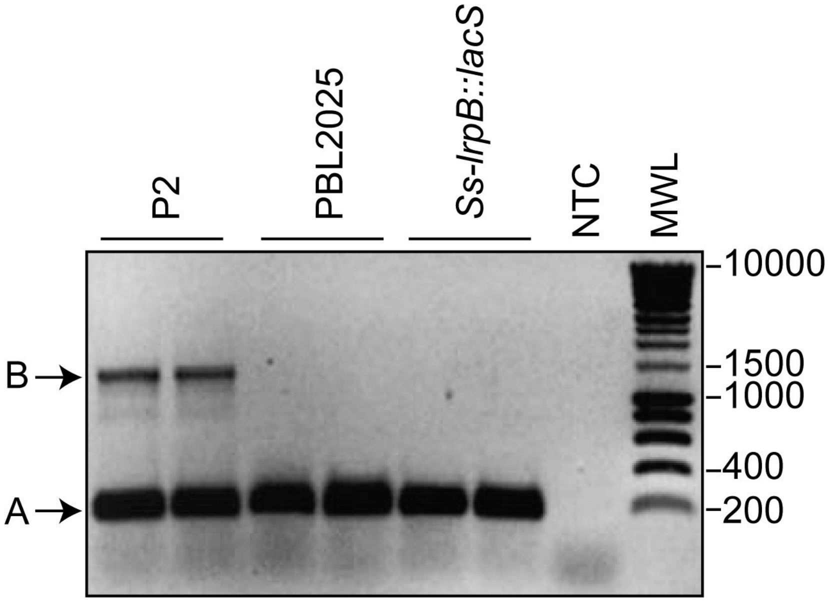

References

Peeters E, Albers SV, Vassart A, Driessen AJM, Charlier D: **Ss-LrpB, a transcriptional regulator from *Sulfolobus solfataricus*, regulates a gene cluster with a pyruvate ferredoxin oxidoreductase-encoding operon and permease genes.** *Molecular Microbiology* 2009, **71**:972–988.

Redder P, Garrett RA: **Mutations and rearrangements in the genome *Sulfolobus solfataricus* P2.** *J Bacteriol* 2006, **188**:4198-4206.

She Q, Singh RK, Confalonieri F, Zivanovic Y, Allard G, Awayez MJ, Chan-Weiher CC, Clausen IG, Curtis BA, De Moors A, *et al*: **The complete genome of the crenarchaeon *Sulfolobus solfataricus* P2.** *Proc Natl Acad Sci USA* 2001, **98**:7835–7840.
