# Supplementary material for: The genome-wide binding profile of the Sulfolobus solfataricus transcription factor Ss-LrpB shows binding events beyond direct transcription regulation
Source: BMC Genomics. 2013 Nov 25;14(1):828. doi: 10.1186/1471-2164-14-828 (PMC4046817; doi:10.1186/1471-2164-14-828)
Supplement: Supplementary file 9 — Additional file 9: Figure S6: Schematic representation of the Ss-lrpB operator structure in the case of absence of ISC1078. (PDF 174 KB) [file 12864_2013_5555_MOESM9_ESM.pdf]

Figure S6. Schematic representation of the *Ss-lrpB* operator structure in the case of absence of ISC1078. The relative position of the insertion site is shown. Box1, Box2 and Box3 are the main operator sites (Peeters *et al.*, 2004) whereas Box4 and Box5 are auxiliary operator sites (Nguyen Duc *et al.*, 2013). The relative position of the newly identified Box6 is given, as is its center-to-center-distance to Box3 and its sequence (inset). The genomic region spanned by the 486-bp DNA fragment tested in EMSA (Fig. 2) is also indicated.

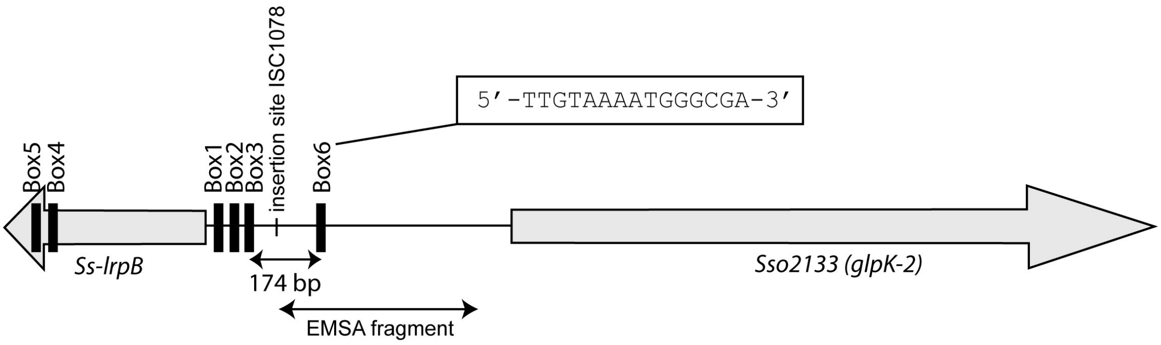

References

Nguyen Duc T, Peeters E, Muyldermans S, Charlier D, Hassanzadeh-Ghassabeh G: **Nanobody(R)-based chromatin immunoprecipitation/micro-array analysis for genome-wide identification of transcription factor DNA binding sites.** *Nucleic Acids Res* 2012, **41**:e59.

Peeters E, Thia-Toong TL, Gigot D, Maes D, Charlier D: **Ss-LrpB, a novel Lrp-like regulator of *Sulfolobus solfataricus* P2, binds cooperatively to three conserved targets in its own control region.** *Molecular Microbiology* 2004, **54**:321–336.
