# Supplementary material for: The genome-wide binding profile of the Sulfolobus solfataricus transcription factor Ss-LrpB shows binding events beyond direct transcription regulation
Source: BMC Genomics. 2013 Nov 25;14(1):828. doi: 10.1186/1471-2164-14-828 (PMC4046817; doi:10.1186/1471-2164-14-828)
Supplement: Supplementary file 10 — Additional file 10: Table S3: In silico predicted Ss-LrpB binding sites (using the binding energy weight matrix) in regions upstream of S. solfataricus P2 ORFs (200 bp upstream of ORF start) with a theoretical KD lower than 5 μM. (PDF 91 KB) [file 12864_2013_5555_MOESM10_ESM.pdf]

Table S3. *In silico* predicted Ss-LrpB binding sites (using the binding energy position weight matrix) in regions upstream of *S. solfataricus* P2 ORFs (200 bp upstream of ORF start) with a theoretical K_D lower than 5 μM. Theoretical K_Ds are calculated based on the binding energy position weight matrix. Ranking is according to predicted binding affinity. N.A. = not applicable.

Locus tag	Gene name	Predicted binding motif	Position (with respect to start codon)	Theo-retical K_D (nM)	Binding detected *in vivo*	Binding tested and detected *in vitro*
Sso0818	N.A.	5'-TTGCAAATTTTTCAA-3'	-160	426		
Sso0902	N.A.	5'-TTGCAGAATATGCAA-3'	-156	845		
Sso7114	*rps27E*	5'-TTGCAAAAGTTACAA-3'	-112	917		
Sso0903	N.A.	5'-TTGCATATTCTGCAA-3'	-183	1010		
Sso0172	N.A.	5'-CTGCACTATTTGCAA-3'	-101	1244		
Sso2128	*porD*	5'-TTGCACATTTTGCCA-3'	-115	1570	x	x
Sso0694	*adkA*	5'-TTGCAGTATTTGCTA-3'	-151	1579		
Sso2131	*Ss-lrpB*	5'-TTGCAAAATTATCAA-3'	-112	1670	x	x
Sso2127	N.A.	5'-TTGCATTTTATGCAA-3'	-104	1768	x	x
Sso2266	*mcmA2*	5'-GTGCAATTATTGCAA-3'	-156	2470		
Sso0628	*purQ*	5'-TTGAAAAAATTGCCA-3'	-73	3084		
Sso0523	N.A.	5'-GTGCCAAATTTGCAA-3'	-95	3218		
Sso0049	N.A.	5'-TTGTAATTTTTTCAA-3'	-72	3309		x
Sso2912	*sat*	5'-GTGCAAAATTTGCAC-3'	-58	3469		
Sso0036	N.A.	5'-CAGAAAAAATTGCAA-3'	-111	3470		
Sso0035	N.A.	5'-TTACAAATTTTTCAA-3'	-196	3529		
Sso2370	N.A.	5'-ATGCAACTATTGCAG-3'	-198	3545		
Sso0483	N.A.	5'-TTGCAGCAAATGCAA-3'	-174	3647		
Sso0448	*hisC*	5'-ATGCAATAACTGCAA-3'	-187	3767		
Sso0639	*argH*	5'-CTGAAAAAATTGCTA-3'	-70	3777		
Sso2438	N.A.	5'-CTGCAATATATGCAT-3'	-130	3788		
Sso2813	N.A.	5'-TTGCAAGACGTGCAG-3'	-148	3854		
Sso2911	*cysH*	5'-GTGCAAATTTTGCAC-3'	-57	4136		
Sso0002	*thiD-1*	5'-TAGTAAGACTTGCAG-3'	-138	4467		
Sso2343	*mtaP*	5'-TTGCAACTATTACAT-3'	-196	4509		x
Ssor01	N.A.	5'-AGGCAATATTTGCAG-3'	-113	4544		
Sso2070	*acsA-7*	5'-CAGCAAATGCTGCAA-3'	-153	4547		
Sso5561	*rps17E*	5'-CTGTAATAATTGCAG-3'	-195	4644		

Sso0441	N.A.	5’-TTGCAATAATTTCAT-3’	-196	4680		
Sso1079	N.A.	5’-ATGCAAAAATTGTAA-3’	-145	4719		
Sso2669	N.A.	5’-TAACAAATATTGCAA-3’	-155	4738		
