# Supplementary material for: The genome-wide binding profile of the Sulfolobus solfataricus transcription factor Ss-LrpB shows binding events beyond direct transcription regulation
Source: BMC Genomics. 2013 Nov 25;14(1):828. doi: 10.1186/1471-2164-14-828 (PMC4046817; doi:10.1186/1471-2164-14-828)
Supplement: Supplementary file 11 — Additional file 11: Figure S7: In vitro binding in the control region of gpT-1 (Sso2342)/ mtaP (Sso2343). (PDF 1 MB) [file 12864_2013_5555_MOESM11_ESM.pdf]

Figure S7. Binding in the control regions of *gpT-1* (*Sso2342*)/*mtaP* (*Sso2343*). A good binding motif has been identified in this region (theoretical KD 7 μM). Whereas no enrichment of this region has been observed in the ChIP-chip experiment, Ss-LrpB forms multiple complexes with DNA containing this sequence *in vitro*. Besides the *in silico* identified site, which is located 196 bp upstream of the TSS, an additional binding motif was predict to be present just upstream of the *gpT-1/mtaP* promoters (theoretical KD 11 μM). This Box displays a direct interaction with Ss-LrpB (see Additional file 12: Figure S8). **A.** Zoomed average ChIP-chip binding profile in the concerned genomic region. Below the profile, the genomic environment is schematically depicted by representing ORFs as grey arrows. The region corresponding to the fragment tested in the *in vitro* binding analysis, is boxed, whereas the center of the identified Ss-LrpB binding site is represented by a vertical bar. **B**. EMSA of binding to a 192 bp fragment encompassing the *gpT-1/mtaP* intergenic region. DNA populations are indicated as follows: free DNA (F), Ss-LrpB-DNA complexes (B1 and B2) and DNA retained in the wells of the acrylamide gel (W). Protein concentrations are given (nM). **C**. Sequence of the *gpT-1/mtaP* intergenic region with indication of putative promoter elements and Ss-LrpB binding site. Coding sequences are shown in uppercase (left-hand side= *gpT-1*, right-hand side = *mtaP*). The TSSs of *gpT-1* and *mtaP*, indicated with bent arrows, were determined by deep sequencing (42) or by primer extension analysis (Additional file 13: Figure S9), respectively.

Reference

Wurtzel O, Sapra R, Chen F, Zhu Y, Simmons BA, Sorek R: **A single-base resolution map of an archaeal transcriptome.** *Genome Res* 2010: **20**:133–141.

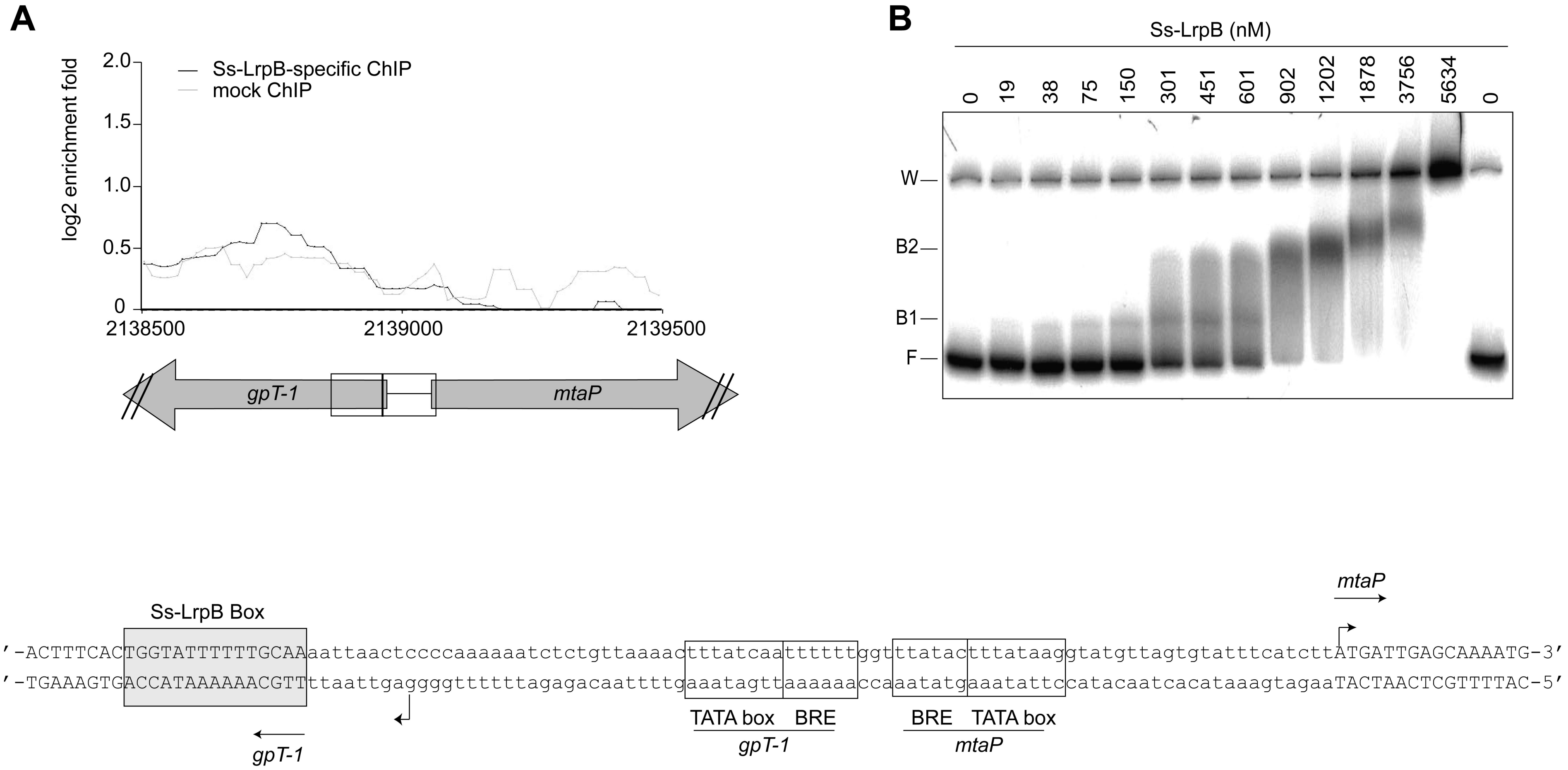
A
Ss-LrpB-specific ChIP
mock ChIP
log2 enrichment fold
2.0
1.5
1.0
0.5
0
2138500
2139000
2139500
gpT-1
mtaP
B
Ss-LrpB (nM)
0
19
38
75
150
301
451
601
902
1202
1878
3756
5634
0
W
B2
B1
F
C
Ss-LrpB Box
mtaP
5'-ACTTTCACTGGTATTTTTTGCAAaattaactccccaaaaaaatctctgttaaaactttatcaattttttggttttatactttataaggtatgttagtgtatttcatcttATGATTGAGCAAAATG-3'
5'-TGAAAGTGACCATAAAAAACGTTttaattgaggggtttttttagagacaattttgaaatagttaaaaaaccaaatatgaaatattccatacaatcacataaagtagaaTACTAACTCGTTTTAC-5'
TATA box
BRE
gpT-1
BRE
TATA box
mtaP
gpT-1
