# Supplementary material for: The genome-wide binding profile of the Sulfolobus solfataricus transcription factor Ss-LrpB shows binding events beyond direct transcription regulation
Source: BMC Genomics. 2013 Nov 25;14(1):828. doi: 10.1186/1471-2164-14-828 (PMC4046817; doi:10.1186/1471-2164-14-828)

Figure S8. *In vitro* binding to the separate Ss-LrpB binding sites identified in the *Sso0049* and *gpT-1/mtaP* control regions. The positions of free DNA (F), Ss-LrpB-DNA complexes (B), single-stranded DNA (SS) and the wells of the acrylamide gel (W) are indicated.

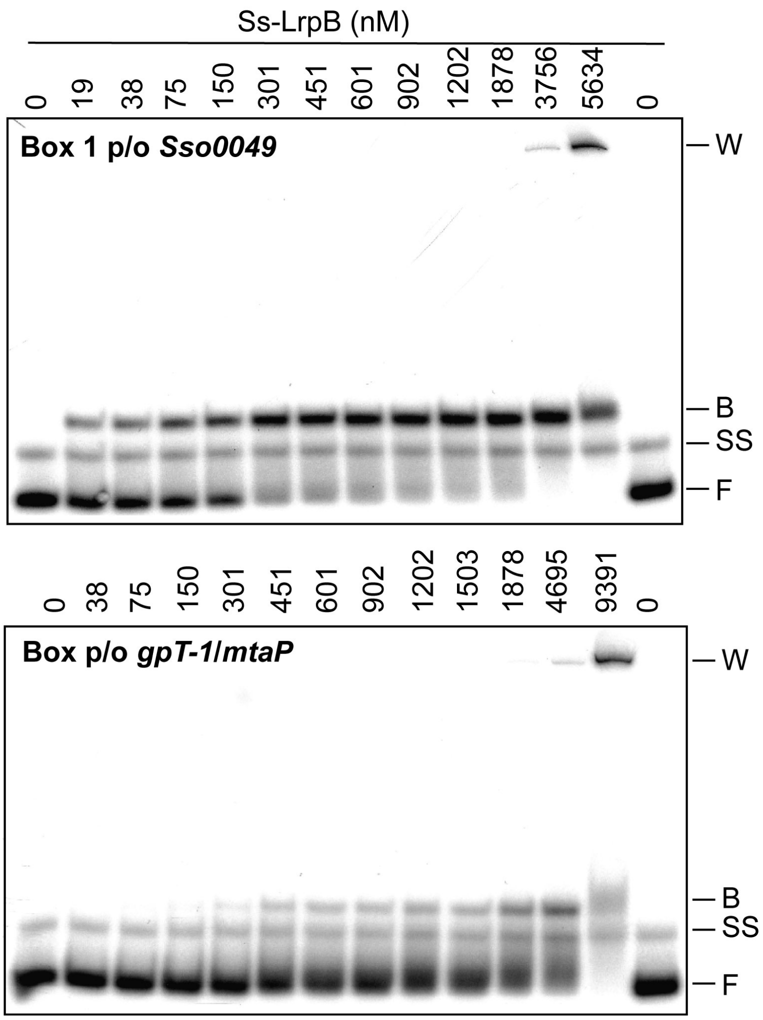

Supplement: Supplementary file 12 — Additional file 12: Figure S8: In vitro binding to the separate Ss-LrpB binding sites identified in the Sso0049 and gpT-1/mtaP control regions. (PDF 300 KB) [file 12864_2013_5555_MOESM12_ESM.pdf]
