# Supplementary material for: The genome-wide binding profile of the Sulfolobus solfataricus transcription factor Ss-LrpB shows binding events beyond direct transcription regulation
Source: BMC Genomics. 2013 Nov 25;14(1):828. doi: 10.1186/1471-2164-14-828 (PMC4046817; doi:10.1186/1471-2164-14-828)
Supplement: Supplementary file 13 — Additional file 13: Figure S9: Primer extension analysis for TSS determination. (PDF 274 KB) [file 12864_2013_5555_MOESM13_ESM.pdf]

Figure S9. Primer extension analysis for TSS determination. This was performed for (**A**) *Sso0049* and (**B**) *Sso2343* (*mtaP)*, genes for which high-affinity Ss-LrpB binding sites have been identified in the respective promoter regions *in vitro* but not *in vivo*. For primer extension experiments, RNA was extracted from a *S. solfataricus* P2 culture during the stationary growth phase using the Trizol reagent and reverse transcriptase primer extension analysis was performed as described before (Enoru-Eta *et al.*, 2002). In each experiment, 100 μg RNA was used. Oligonucleotides (Sigma-Aldrich) that were used for the primer extension analyses are given in Additional file 2: Table S1. Reference ladders were generated by chemical sequencing (Maxam & Gilbert, 1980). TSSs, determined by this experiment, are indicated on the corresponding sequences in Figure 5D and Additional file 11: Figure S7C. Adjustments were made since the DNA fragment migrates 1.5 positions higher as compared to the sequence ladder, explained by the molecular differences on the 3' end residues generated by chemical sequencing on the one hand or the reverse transcription reaction on the other hand.

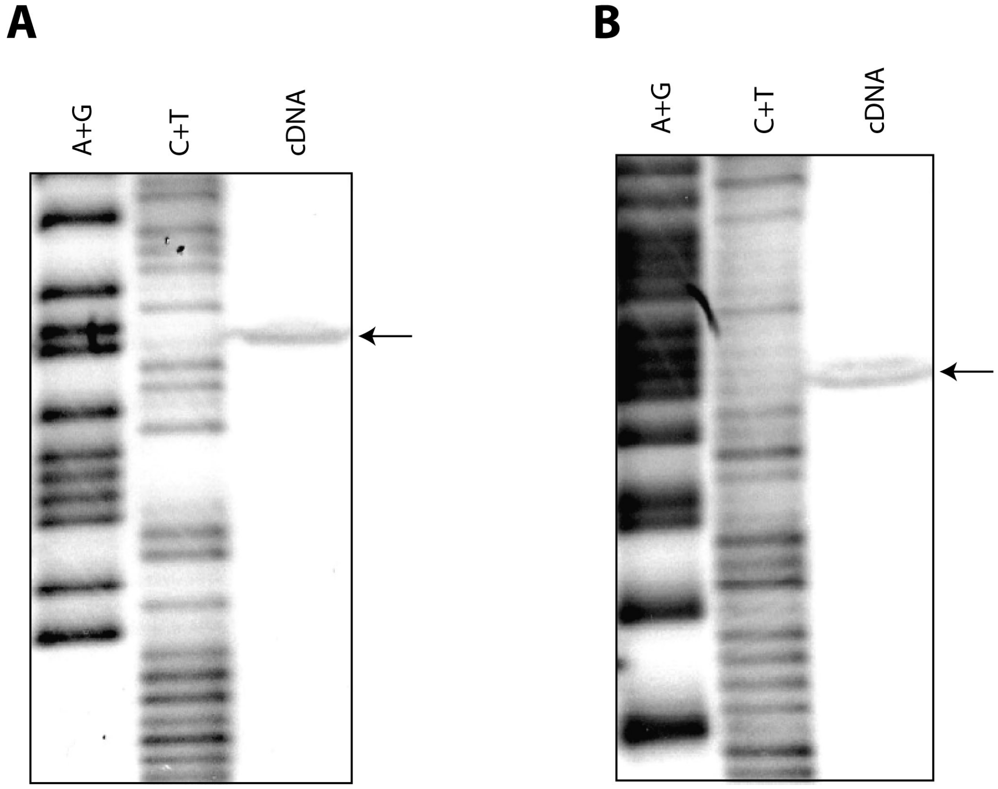

References

Enoru-Eta J, Gigot D, Glansdorff N, Charlier D: **High resolution contact probing of the Lrp-like DNA-binding protein Ss-Lrp from the hyperthermoacidophilic crenarchaeote *Sulfolobus solfataricus* P2.** *Molecular Microbiology* 2002, **45**:1541–1555.

Maxam AM, Gilbert W: **Sequencing end-labeled DNA with base-specific chemical cleavages.** *Meth Enzymol* 1980, **65**:499–560.
